# Wireless implantable optical probe for continuous monitoring of oxygen saturation in flaps and organ grafts

**DOI:** 10.1038/s41467-022-30594-z

**Published:** 2022-05-30

**Authors:** Hexia Guo, Wubin Bai, Wei Ouyang, Yihan Liu, Changsheng Wu, Yameng Xu, Yang Weng, Hao Zang, Yiming Liu, Lauren Jacobson, Ziying Hu, Yihang Wang, Hany M. Arafa, Quansan Yang, Di Lu, Shuo Li, Lin Zhang, Xun Xiao, Abraham Vázquez-Guardado, Joanna Ciatti, Elizabeth Dempsey, Nayereh Ghoreishi-Haack, Emily A. Waters, Chad R. Haney, Amanda M. Westman, Matthew R. MacEwan, Mitchell A. Pet, John A. Rogers

**Affiliations:** 1 Department of Materials Science and Engineering, Northwestern University, Evanston, IL 60208 USA; 2 Querrey Simpson Institute for Bioelectronics, Northwestern University, Evanston, IL 60208 USA; 3 Department of Applied Physical Sciences, University of North Carolina at Chapel Hill, Chapel Hill, NC 27514 USA; 4 The Institute of Materials Science and Engineering, Washington University in St. Louis, St. Louis, MO 63110 USA; 5 Division of Plastic and Reconstructive Surgery, Department of Surgery, Washington University School of Medicine, St. Louis, MO 63110 USA; 6 Department of Mechanical Engineering, Northwestern University, Evanston, IL 60208 USA; 7 Developmental Therapeutics Core, Northwestern University, Evanston, IL 60208 USA; 8 Center for Advanced Molecular Imaging, Northwestern University, Evanston, IL 60208 USA; 9 Department of Neurosurgery, Washington University School of Medicine, St. Louis, MO 63110 USA; 10 Department of Biomedical Engineering, Northwestern University, Evanston, IL 60208 USA; 11 Department of Chemistry, Northwestern University, Evanston, IL 60208 USA; 12 Department of Neurological Surgery, Feinberg School of Medicine, Northwestern University, Evanston, IL 60208 USA

**Keywords:** Biomedical engineering, Implants, Optoelectronic devices and components, Diagnosis, Translational research

## Abstract

Continuous, real-time monitoring of perfusion after microsurgical free tissue transfer or solid organ allotransplantation procedures can facilitate early diagnosis of and intervention for anastomotic thrombosis. Current technologies including Doppler systems, cutaneous O_2_-sensing probes, and fluorine magnetic resonance imaging methods are limited by their intermittent measurements, requirements for skilled personnel, indirect interfaces, and/or their tethered connections. This paper reports a wireless, miniaturized, minimally invasive near-infrared spectroscopic system designed for uninterrupted monitoring of local-tissue oxygenation. A bioresorbable barbed structure anchors the probe stably at implantation sites for a time period matched to the clinical need, with the ability for facile removal afterward. The probe connects to a skin-interfaced electronic module for wireless access to essential physiological parameters, including local tissue oxygenation, pulse oxygenation, and heart rate. In vitro tests and in vivo studies in porcine flap and kidney models demonstrate the ability of the system to continuously measure oxygenation with high accuracy and sensitivity.

## Abstract (web summary)

Although continuous monitoring of tissue oxygenation is critically important after tissue/organ graft procedures, current technologies have key limitations. Here, the authors develop a miniaturized, minimally invasive, self-anchoring optical probe and demonstrate continuous monitoring of oxygenation in porcine flap and organ models.

## Introduction

Continuous monitoring of regional tissue oxygenation (StO_2_) at depth represents a critical clinical need for microsurgical free tissue transfer and solid organ allotransplantation. Prompt detection of changes allows immediate identification and emergent operative intervention in cases of tissue ischemia resultant from early post-operative anastomotic thrombosis (76–91% appears within 48 h and 94–97% within a week)^1,2^. Existing technologies that are widely used in clinical settings for monitoring free flaps include internal Doppler systems for flow^3,4^ (such as Synovis Flow Coupler, Baxter Inc.^5^, and Cook-Swartz Doppler, Catalent Inc^6^.) and near-infrared spectroscopy for oxygenation (NIRS, such as T.Ox, ViOptix Inc.)^7,8^. The former monitors blood flow in an artery or vein with a piezoelectric probe placed directly adjacent to the anastomosis. The response, however, does not directly reflect muscular oxygenation, and the device deployment introduces risks of mechanical disruption of the anastomosis. The latter offers continuous monitoring of oxygenation, but the interface to the flap relies on a cutaneous paddle, which limits its applicability. Moreover, both systems require electrical wires tethered to external, benchtop hardware, which decreases user comfort and introduces additional complications and risks^7,9^.

Allotransplanted solid organs are often more critical than microsurgical free flaps, as anastomotic thrombosis in these cases leads to organ dysfunction and necrosis which can even result in patient death^10^. Tissue oxygenation levels of internal organs reflect metabolic functions and health status, thus serving as an essential biomarker for monitoring recovery processes during organ transplantations, especially for kidney or liver transplantation^10–13^. In particular, continuous monitoring of internal organs and tissues can provide a means for early determination of vascular complications, including stenosis and thrombosis, which, if not treated at an early stage, could lead to graft dysfunction, and loss post-transplant^10,11,14–16^. Existing clinically available systems involve electrochemical and luminescence sensors, fiber optic probes, electron paramagnetic resonance spectroscopy techniques, or fluorine magnetic resonance imaging methods^17–20^. These systems, however, may yield inaccurate measurements, all require tethering connections, and most experience delayed response time for a single measurement, all of which limit their potential for real-time monitoring﻿^17^. Skin-mounted systems can determine oxygenation continuously, but the limited penetration depth precludes applicability to organs or regional tissues at depth.

Recent advances in wireless technologies^21–24^ and bio-integrated electronic systems^25–27^ offer promising approaches for monitoring oxygenation globally or locally^22,23,26,28–31^. Several challenges, however, limit their practical utilization in continuous oxygenation monitoring of local tissue. A subdermally implantable wireless oximeter reported by Zhang et al.^22^ measures absolute changes in hemoglobin and deoxyhemoglobin accurately via a spectroscopic method, but an approximation of initial total hemoglobin concentration is necessary to calculate regional tissue oxygen saturation. Furthermore, requirements for wireless power harvesting via magnetic resonant coupling limit operation to within a short distance of a transmissing antenna. Alternative approaches that use ultrasonics as a power transfer mechanism overcome the distance limitation but requirements for skin-mounted ultrasound transducers oriented toward the implants introduce practical challenges in clinical settings^26^. A wireless implantable oximeter for measuring cardiac pulse oxygen saturation (SpO_2_)^23^ and a wireless skin-interfaced sensor for monitoring cerebral hemodynamics^30^ suggest strategies for monitoring local deep-tissue StO_2_ in which near-infrared spectroscopic (NIRS) probes with light sources and multiple photodiodes at various distances provides the basis for spatially resolved spectroscopic measurements of absorbance for calculation of tissue StO_2_^32^.

This paper reports a wireless, implantable optoelectronic microsystem of this general type, configured for continuous monitoring of StO_2_ of regional tissues at targeted depths. Deployments in a porcine model illustrate various operational and clinically relevant performance aspects of this technology. The results demonstrate the potential to overcome key limitations associated with existing technologies for tissue oxygenation. The main advances include: (1) an injectable, flexible probe that supports a pair of microscale inorganic light-emitting diodes and photodetectors (μ-ILEDs and μ-IPDs), designed for minimally invasive implantation; (2) a spatially resolved NIRS scheme that enables measurement of the gradient of absorbance for precise calculation of oxygenation levels across various tissues; (3) a collection of bioresorbable barbs distributed along the length of the probe to enable mechanically stable anchoring to adjacent soft tissues during a desired operating period and to facilitate removal without tissue damage, after complete bioresorption. A small, wireless electronic module based on Bluetooth Low-Energy (BLE) protocols supports data communication with a graphical user interface (GUI) on a separate device. This module attaches to the skin and connects to the implantable probe via a hard-wired interface. In vivo studies using porcine flap and kidney models of arterial and venous occlusion demonstrate the ability of this device to capture changes in tissue oxygenation during ischemia and congestion in a real-time manner. These experiments suggest possible applications in continuous monitoring of tissue oxygenation in clinical settings, such as for various acute models and procedures including those in free muscle-flap transfer and organ transplantation. Long-term functional testing in vitro also demonstrates the potential of the device for long-term applications in semi-chronic models^22,33^. Compared to the wireless miniaturized StO_2_ monitoring systems developed by Zhang et al.^22^ and the partial pressure of oxygen monitoring system developed by Sonmezoglu et al.^26^, the system introduced here avoids the need for near-field magnetic coupling schemes or for ultrasonic power delivery strategies, both of which limit patient mobility.

## Results

### Design and features of the wireless, implantable NIRS optoelectronic microsystem

**Fig. 1:**
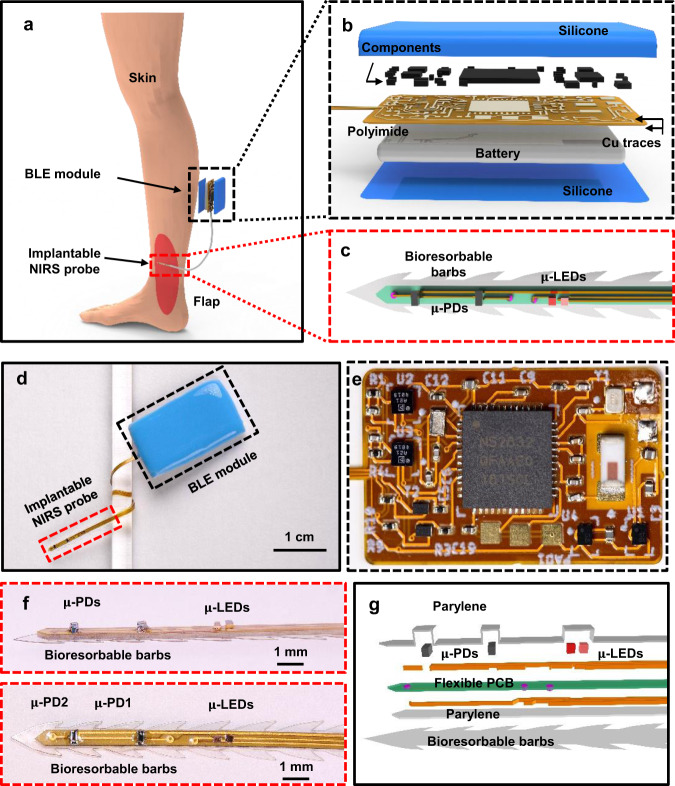
Wireless, implantable near-infrared spectroscopic (NIRS) probing system for local-tissue oximetry. **a** Schematic illustration of a NIRS probe and wireless module depolyed in a microsurgically transferred free muscular flap. **b** Exploded view of the wireless communication module based on Bluetooth Low Energy (BLE) technology. **c** Top-down view of the sensing probe featuring the bioresorbable barbs and sensing units. **d** Image of a wireless NIRS system with sensing probe wrapped around a plastic tube to highlight its mechanical flexibility. **e** Enlarged view of the wireless communication module supported on a flexible printed circuit board. **f** Images of the sensing probe, top: side view, bottom: top-down view. **g** Exploded view of the sensing probe with a conformal layer of parylene for encapsulation and a bioresorbable base layer consisting of distributed bioresorbable barbs for temporarily anchoring the probe to the surrounding soft tissues.

The design of the system includes: (i) a sensor probe with two μ-ILEDs (length 340 μm, width 340 μm, thickness 270 μm) and two μ-IPDs (length 670 μm, width 300 μm, thickness 280 μm) interconnected by a flexible printed circuit board (PCB), (ii) bioresorbable barbs made of poly(lactic-co-glycolic acid) (PLGA, thickness 50 μm, total barb depth 300 μm, barb angle 54°, throat angle 22°, throat length 1.30 mm, spacing 1.35 mm. without kick-up), that extend along the edges of the probe, and (iii) a small, battery-powered module that controls the system and provides wireless data communication using BLE protocols (Fig. 1). A thin encapsulation layer (parylene, thickness 14 μm) prevents penetration of biofluids into the electronic components and ensures stable device performance. Figure 1a presents a schematic illustration that uses a flap model to demonstrate the potential use of this device in flap surgery. Figure 1b, c presents schematic illustrations of the electronics module and the sensor probe.

Figure 1d shows an image of the overall device, highlighting the flexibility of the implantable NIRS probe. Figure 1e presents an enlarged view of the BLE module (block diagram in Fig. S1a), the components of which include: (i) a BLE microcontroller (NRF52832, Nordic Semiconductor, Inc.) for activating the μ-ILEDs in a low-duty cycle mode (frequency 50 Hz, 10% duty cycle) for processing the data, and for communicating wirelessly with a computer or mobile device; (ii) two analog-digital converters (ADC) for signal sampling at 100 Sa/s for each μ-IPD; (iii) two transimpedance amplifiers with corresponding feedback resistors of 1MΩ for μ-IPD1 and 4.3 MΩ for μ-IPD2 (as labeled in Fig. 1f), and (iv) a 40 mAh rechargeable Li-ion battery that allows for 40 h of operation from a single charge. A customized graphical user interface (GUI) developed in Python supports real-time data display and storage (Fig. S1b). Figure S1c presents a flow chart for determining tissue oxygenation saturation (StO_2_), pulse oxygenation saturation (SpO_2_), and heart rate (HR) from the measured data. Details appear in the Methods sections.

Figure 1f presents a magnified side-view and a top-view of the probe. The emission wavelengths of the μ-ILEDs are 660 nm and 850 nm. For the red LED, the forward voltage, maximum reverse current, output power, and peak wavelength are 2.1 V, 10 µA, 15 mW, and 660 nm, respectively. For the NIR LED, the forward voltage, maximum reverse current, output power, and peak wavelength are 1.55 V, 10 µA, 6 mW, and 850 nm, respectively. Figure S2 shows the characteristics of the photodetectors. The distances between the μ-ILEDs and the two photodetectors are 4 mm (μ-IPD1) and 7 mm (μ-IPD2), selected by considering the probing volume and signal quality (Figs. S2–3)^28,34,35^. Measurements at various distances (ranging from 1 to 11 mm) inside a flap of porcine muscle enables a basic optimization of the sensitivity of the probe. The measurements (Fig. S3d) indicate a decrease in the signal with an increase of the distance. The signal-to-noise ratio increases dramatically when the distance is larger than 8 mm, mostly due to reduced signals and increased heterogeneity of the sensing volume. Figure 1g shows an exploded view illustration of the probe with the μ-ILEDs and μ-IPDs, a transparent, biocompatible encapsulation layer of parylene (14 μm) coated onto the device as a biofluid barrier, and a set of bioresorbable barbs on a substrate of PLGA at the bottom side of the probe.

### Electrical, optical, and thermal characteristics

Deoxyhemoglobin (Hb) and oxyhemoglobin (HbO_2_) have distinct absorption spectra^32,36^, such that optical measurements of hemoglobin-containing tissues at selected wavelengths establish the basis for calculating tissue oxygenation, using well-established procedures. The emission spectra in Fig. S2a show peak wavelengths of 660 nm and 850 nm, both within a window of relative transparency for biological tissues that allows deep-tissue penetration^36,37^. These wavelengths lie above and below the isosbestic point (~805 nm) to allow sensitive measurements via differences in absorption between HbO_2_ and Hb^38^. The efficiencies of the μ-ILEDs are 25% and 20% at 660 and 850 nm, respectively, as shown in the data in Fig. S2b.

**Fig. 2:**
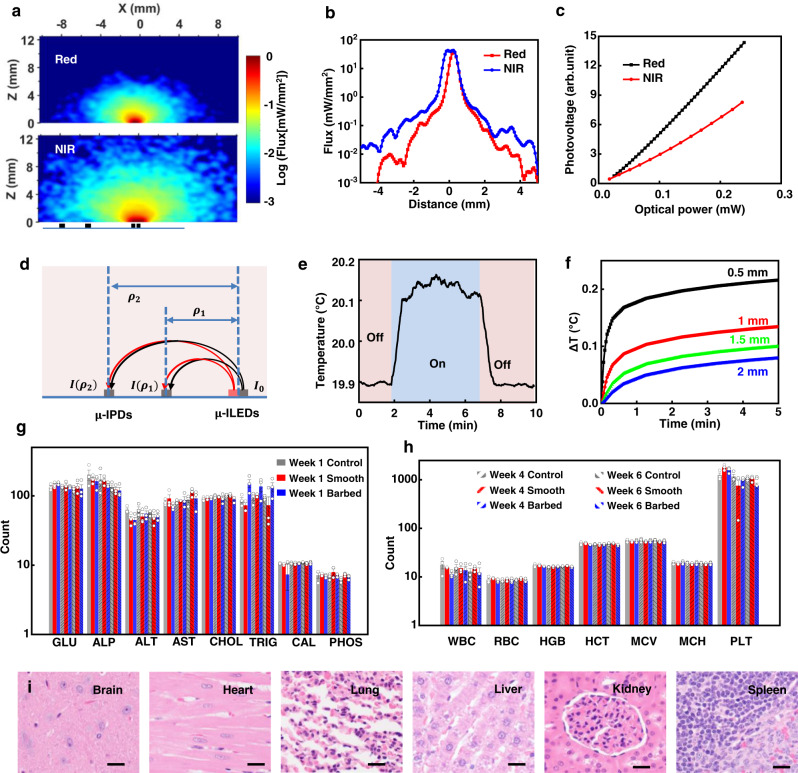
Characterization of the NIRS probe. **a** Monte Carlo simulation results for the spatial distribution of the normalized flux intensity of red (top) and NIR (bottom) light emitted from the μ-ILEDs on the probe into muscle tissue. **b** Simulated flux distribution along the probe direction, with *x*-axis as defined in **a**. **c** Measured photovoltage response of the μ-IPD as a function of illumination intensity. **d** Schematic illustration of the design parameters for the spatially resolved spectroscopic method with a pair of μ-IPDs and a pair of μ-ILEDs. **e** Measured temperature variations at regions near the μ-ILEDs during normal operation (50 Hz, 10% duty cycle of red and NIR μ-LEDs) for 5 min in porcine meat. **f** Finite Element Analysis (FEA) results for the change in temperature during normal operation, at different distances above the μ-ILEDs. **g**, **h** Measured results for **g** blood chemistry and **h** complete blood count of rats with sham surgery (control) and implanted NIRS probes (two groups, one with bioresorbable barbs, the other without bioresorbable barbs) for 1, 4, and 6 weeks, respectively. GLU glucose (mg/dL), ALP alkaline phosphatase (U/L), ALT alanine aminotransferase (U/L), AST aspartate transaminase (U/L), CHOL cholesterol (mg/dL), TRIG triglycerides (mg/dL), CAL calcium (mg/dL), PHOS phosphorus (mg/dL), WBC white blood cell (×1000/μL), RBC red blood cell (×1,000,000/μL), HGB blood hemoglobin level (g/dL), HCT hematocrit level (%), MCV mean corpuscular volume (fL), MCH mean corpuscular hemoglobin (pg), PLT platelet count in blood (×1000/μL). *n* = 3 independent samples. All data are shown as mean ± SEM. **i** Images of tissue sections of brain, heart, lung, liver, kidney, and spleen, 6-weeks post-implantation of a barbed NIRS probe. All tissue sections were stained with hematoxylin and eosin (H&E) before imaging. H&E, 400×. Scale bar: 25 μm. Source data are provided as a Source Data file.

Monte Carlo methods with optical properties of human tissues of flap muscles^32,39^ reveal features of light propagation at these wavelengths^40^. Figure 2a provides spatial distributions of the illumination and quantitative insights. Further details appear in the Methods. The emission intensity profiles (Fig. 2b: *x*-axis, and Fig. S3a: *z*-axis) and cross-sectional planes across the μ-ILEDs show the penetration depths, defined as the position where the optical irradiance decreases to 10^−3^ mW/mm^2^, are 4.12 and 6.17 mm, corresponding to volumes of 95.3 and 179.8 mm^3^, for the red and NIR wavelengths, respectively (Fig. S3b, c). The results are approximately consistent with experimental measurements (Fig. S3d) using devices implanted in a porcine flap. The findings provide design guidelines tailored to different clinical needs, ranging from muscle and skin flaps to internal organs such as the kidney and liver.

Figure 2d presents a schematic illustration of the principle of spatially resolved spectroscopy with two photodetectors and design parameters optimized for measuring StO_2_. The Methods section provides detailed procedures for calculating the light absorption coefficients *μ*_*a*_ at the emission wavelengths of the μ-ILEDs (*λ*_1_ and *λ*_2_), based on the gradient of absorbance $\frac{\partial A}{\partial \rho }$, where ${A}_{{\lambda }_{1{or}2},{\rho }_{1{or}2}}$ are absorbances at *λ*_1_ and *λ*_2_, respectively, and at distances, *ρ*_1_ and *ρ*_2_, respectively, from the light source. Thus, the StO_2_ level can be accurately calculated from:

1 $$\left[\begin{array}{c}k{C}_{{Hb}}\\ k{C}_{{Hb{{{{{\mathrm{O}}}}}2}}}\end{array}\right]=\frac{1}{{{{{{\rm{ln}}}}}}\left(10\right)}{\left[\begin{array}{cc}{\varepsilon }_{{Hb},{\lambda }_{1}} & {\varepsilon }_{{Hb{{{{{\mathrm{O}}}}}2}},{\lambda }_{1}}\\ {\varepsilon }_{{Hb},{\lambda }_{2}} & {\varepsilon }_{{Hb{{{{{\mathrm{O}}}}}2}},{\lambda }_{2}}\end{array}\right]}^{-1}\left[\begin{array}{c}k{\mu }_{a,{\lambda }_{1}}\\ k{\mu }_{a,{\lambda }_{2}}\end{array}\right]$$

2 $${{{{{\mathrm{{St}}}}}}}{{{{{\mathrm{{O}}}}}_{2}}}=\frac{{C}_{{Hb{{{{{\mathrm{O}}}}}2}}}}{{C}_{{Hb{{{{{\mathrm{O}}}}}2}}}+{C}_{{Hb}}}=\frac{{{kC}}_{{Hb{{{{{\mathrm{O}}}}}2}}}}{{{kC}}_{{Hb{{{{{\mathrm{O}}}}}2}}}+k{C}_{{Hb}}}$$

Where *k* is an unknown constant but is canceled out when calculating StO_2_, and ${\varepsilon }_{Hb\,or\,Hb{{{{{{\mathrm{O}}}}}_{2}}},{\lambda }_{1\,or\,2}}$ are the specific molar extinction coefficients corresponding to the wavelengths *λ*_1_ and *λ*_2_, respectively, and deoxygenated and oxygenated blood, repectively. Based on the theory of electromagnetic radiation energy transfer in the limit of a semi-infinite medium, the absorption coefficient *kμ*_*a*_, which is directly related to the concentration of Hb and HbO_2_, can be measured from the gradient of absorbance $\frac{\partial A}{\partial \rho }$. The multi-wavelength and multi-source-detector distance design enables calculations of StO_2_ based on measurements from the probe itself. In contrast, designs that use only one μ-IPD^22,23,41^ require a baseline calibration with the initial StO_2_ and total hemoglobin concentration as shown in Eq. 3. This scheme uses the modified Beer-Lambert law to determine Δ*C*_*Hb*_ and Δ*C*_*HbO*2_ with an external calibration for total hemoglobin concentration and an initial value of StO_2_ to determine StO_2_ (*t*), both typically obtained by estimation or measurement of reference materials. The use of two μ-IPDs, as described here, supports spatially resolved spectroscopy with improved accuracy in the determination of StO_2_ by eliminating the need to approximate the initial total hemoglobin concentration or the initial StO_2_.

3 $${{{{{\mathrm{{St}}}}}{O}_{2}}}\left(t\right) 	=\frac{{C}_{{Hb{{{{{\mathrm{O}}}}}2}}}(t)}{{C}_{{Hb{{{{{\mathrm{O}}}}}2}}}(t)+{C}_{{HHb}}(t)}\\ 	=\frac{{C}_{{Hb},t}* {{{{{\mathrm{{St}}}}}{O}_{2}}}\left(t=0\right)+\Delta {C}_{{Hb{{{{{\mathrm{O}}}}}2}}}(t)}{{C}_{{Hb},t}* {{{{{\mathrm{{St}}}}}{O}_{2}}}\left(t=0\right)+\Delta {C}_{{Hb{{{{{\mathrm{O}}}}}2}}}(t)+{C}_{{Hb},t}* (1-{{{{{{\mathrm{St}}}}}}}{{{{{\mathrm{{O}}}}}_{2}}}\left(t=0\right))+\Delta {C}_{{Hb}}(t)}$$

The operation of the two μ-ILEDs involves currents of 1.7 mA and 3.0 mA, corresponding to optical powers of 0.77 mW and 0.58 mW, for red and NIR μ-ILEDs, respectively. A 10% duty cycle reduces power consumption and minimizes heat generation, as demonstrated both experimentally and computationally (Fig. S4a). As illustrated in Figs. 2e and S4b, c, operating a device in porcine meat or on the skin for 5 min, leads to steady-state increases in temperature of ~0.2 °C, which represents minimal risk^42,43^. Increases in temperature in tissue adjacent to the probe can be determined by finite element analysis (FEA), as shown in Figs. 2f and S4d. For an initial value of 19.9 °C, the peak temperature reaches ~20.3 °C after 5 min. All four locations evaluated in these simulations show increases that are less than 0.25 °C, whereas distances larger than 1.5 mm have increases in temperature less than 0.1 °C. The steady-state increase of temperature in porcine meat at various duty cycles especially at 100% duty cycle (Fig. S4e), suggests that in the case of tissues with significant light attenuation, the optical power can be further increased, with a sampling rate as low as once per minute^44,45^.

Rat models serve as the basis for evaluating the biocompatibility of probes with and without bioresorbable barbs, as in Figs. 2g–i and S5–7. The findings reveal no signs of toxicity during a 6-week study with groups of animals that received sham surgeries (control), those groups that received implantation of probes without bioresorbable barbs (smooth), and those that received implantation of probes with bioresorbable barbs (barbed). At the end of the study period, the body weight and the weight of major organs (heart, lung, liver, kidney, spleen, and brain) from the smooth and barbed groups show no significant differences from the control group (Fig. S5). Analysis of blood chemistry and complete blood counts (Figs. 2g, h and S6a, b) at 1, 4, and 6 weeks also show no significant differences, consistent with no changes in electrolyte or enzyme balance, and no signs of organ injury or damage^46,47^. Histopathologic evaluation of the examined major organs (Figs. 2i and S6c) reveals no evidence of systematic toxicity. Minor skin inflammation and fibrosis appear microscopically at the implantation sites with similar incidence and severity across all groups, which is common in typical surgical procedures (Fig. S7). Additional details appear in Supplementary Note 1.

### Bioresorbable barbs

**Fig. 3:**
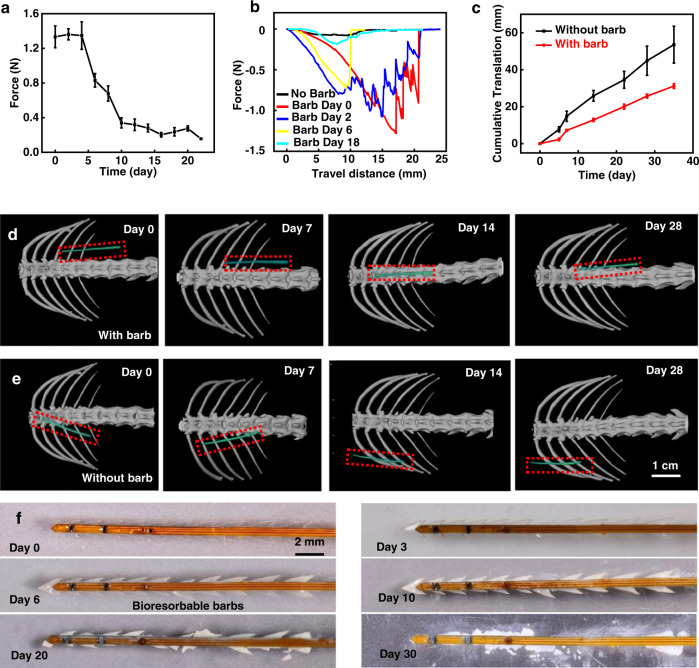
Characterization of NIRS probes with bioresorbable barbs. **a** Measured maximum pulling force to remove a probe inserted in a tissue phantom for different times of immersion in phosphate buffered saline (PBS) solution at 37 °C. *n* = 3 independent samples. All data are shown as mean ± SEM. **b** Representative pulling force as a function of removal distance for probes with and without barbs. **c** Cumulative translation distance of NIRS probes with and without barbs implanted in vivo. The measurement relies on quantitative analysis of Computed Tomography (CT) images of NIRS probes implanted subcutaneously in mice. *n* = 3 independent samples. All data are shown as mean ± SEM. **d**, **e** CT images showing the location of implanted NIRS probes with or without barbs respectively. All images share the same scale bar. **f** Images showing the process of bioresorption of barbs during soaking tests in PBS at 37 °C. Source data are provided as a Source Data file.

Previously reported designs for catheter-type oxygenation sensors exploit fine sutures to mechanically stabilize the probe at the implantation site^23^, This approach can be useful, but it introduces risks in tissue damage and it can only be applied in scenarios that present surgical access. Here we introduce a self-anchoring barbed structure^48^ constructed with a bioresorbable polymer that extends around the probe. This structure automatically secures the probe to surrounding soft tissue during the process of insertion to the targeted site. Figures 1c, f–g, and S11 present the structure and design considerations (Supplementary Note 2) for barbs made of poly(lactic-co-glycolic acid) (PLGA, 50:50), an FDA-approved bioresorbable polymer. The thickness, composition, and molecular weight can be selected to undergo dissolution by hydrolysis reactions with biofluids for lifetimes matched to the clinical monitoring need^49,50^. After resorption, the probe can be easily removed from the tissue. Figure 3a, b presents representative profiles of the pulling force required to remove barbed probes from porcine meat and the change of the maximum pulling force as a function of time of immersion in phosphate buffer saline solution (PBS) at 37 °C.

The probe is compatible with and visible under conventional imaging technologies including X-ray computed tomography (Micro-CT). Figure 3c summarizes the cumulative translation distance relative to the spine for both types of devices, where the probe with barbs shows minimal movement until day 7, consistent with the rate of bioresorption of the barbs (Fig. 3a). Here, the probe with barbs on average moves ~0.46 mm/day during the first 5 days, and the cumulative translation is about 2.3 mm, which is comparable to the distance between optical components in the probe and much smaller than typical tissue sizes (such as porcine flap and kidney, about 10 × 5 × 2 cm). Therefore, the movement has minimal impact on the measurement. During day 5–7, the barbs start to soften, consistent with a significant drop in the pulling force associated with removal (Fig. 3a, f). Correspondingly, the probe with barbs begins to increase in its movement, as in Fig. 3c. In Fig. 3d, e, a series of CT images at various stages of implantation inside subcutaneous regions of rat models further demonstrate the effectiveness of the barbed structure in mechanical stabilization. Implanted probes in the subcutaneous tissue of freely moving rats show negligible changes in location and orientation until significant bioresorption occurs at 10–14 days post-implantation. By comparison, probes without the barbed structures show significant rotations, translations, and other types of movement throughout the period of observation. Figure 3f further confirms the resorption and the change of mechanical properties of PLGA barbs at 37 °C in PBS. The barbs begin to soften and change in appearance from transparent to white after soaking in PBS for 6 days. Significant disintegration occurs by day 10, thereby facilitating extraction.

### In vivo demonstration in a porcine flap model

Studies of device performance and reliability involve comparisons to results from a commercial blood gas analyzer (Abbott i-SAT CG8+)^51^ through in vitro tests using fresh defibrinated horse blood at a series of oxygenation levels. As shown in Fig. S8, oxygenation measurements with the device introduced here are consistent with those of the commercial platform. Figure S9 shows the effective signal intensity with sufficient signal-to-noise ratio of the NIRS probe for deployment inside various porcine organs, including the stomach, kidney, small intestine, liver, and heart (Fig. S10), indicating its applicability across various tissues.

**Fig. 4:**
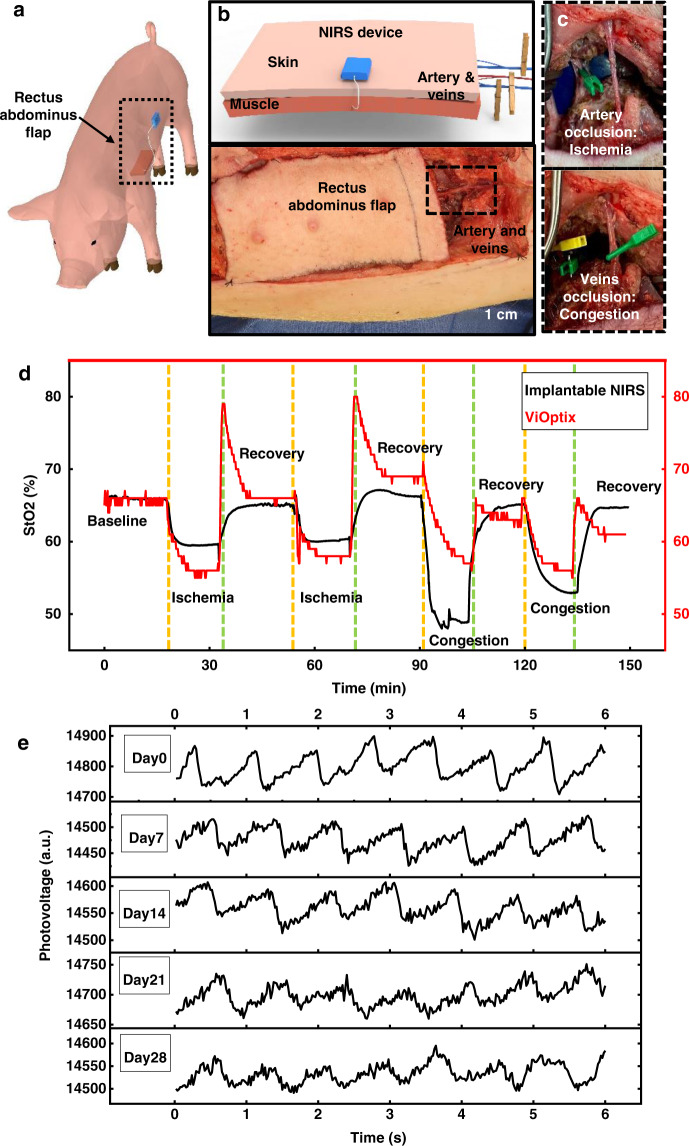
In vivo measurements in a porcine flap model. **a**, **b** Schematic illustrations of the implantation strategy using the left rectus abdominus flap in a porcine model. **c** Image of the occlusion of arteries and veins to simulate events of ischemia and congestion, respectively. **d** Measured tissue oxygenation saturation from the implantable NIRS probe and from a skin-mounted device (ViOptix) during multiple experimental cycles that simulate events of ischemia and congestion. **e** Measured patterns of pulsation recorded from the fingertips using a NIRS probe after immersion in phosphate buffered saline solution at 37 °C for various time periods. Source data are provided as a Source Data file.

**Fig. 5:**
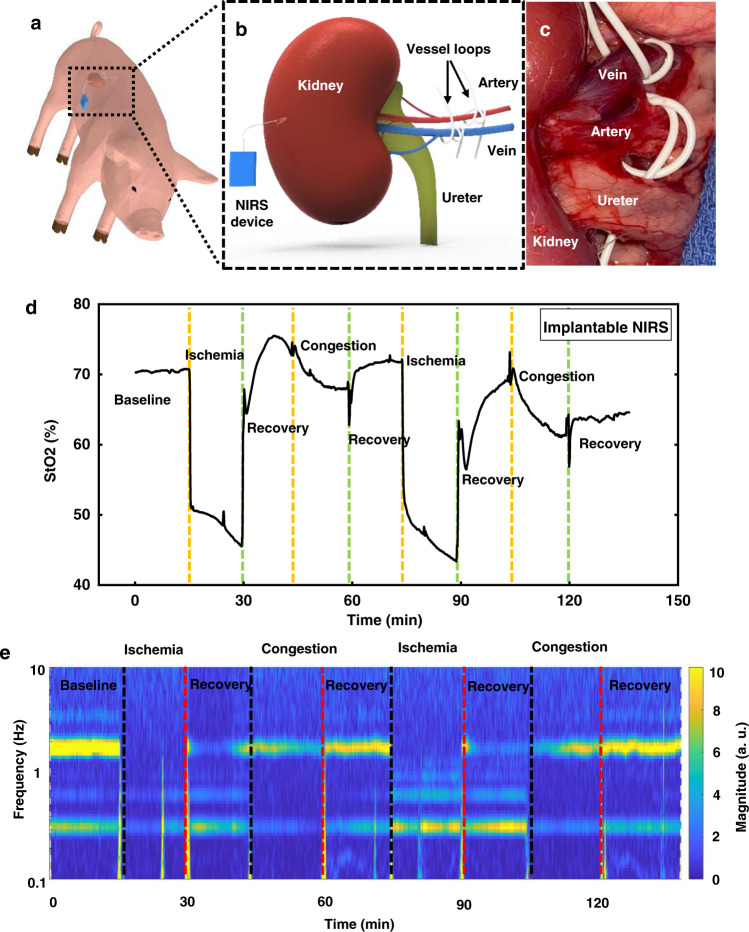
In vivo measurements in a porcine kidney model. **a**, **b** Schematic illustrations of the implantation strategy of the NIRS probes in the porcine kidney model. **c** Image of the occlusion of arteries and veins to simulate events of ischemia and congestion, respectively, with vessel loops. **d** Measured tissue oxygenation saturation using the implantable NIRS probe through multiple experimental cycles of ischemia and congestion. The timeline of respective events (ischemia, congestion, and recovery) shows a consistent match with that of occlusion and release of arteries and veins. **e** Measured signals plotted as spectrograms to highlight variations of pulsation patterns and respiration patterns. The timelines match well with those of the occlusion and release events, respectively. Source data are provided as a Source Data file.

The use of the probes in large animal models validates the potential for clinical application with human subjects. Here, two porcine models, a muscle-flap model and a kidney model (Figs. 4–5) enable systematic evaluations of the devices in monitoring StO_2_ at targeted locations in response to simulated adverse events (ischemia and congestion).

The surgical procedures for raising a flap and implanting the device appear in Methods. An innovative deployment method using a peel-away sheath could be explored in the future to achieve minimally invasive implantation (Fig. S12). As there is no commercially available system for continuous monitoring of StO_2_ in muscle, a skin-mounted sensor (ViOptix) was adopted for simultaneous measurement to yield comparison data^7,52^. The initial part of the test involves stabilizing the flap for 15 min during a baseline period. Subsequently, an Acland clamp applied to a deep superior epigastric artery induces complete ischemia; application to superficial and deep superior epigastric veins induces venous congestion. Releasing the clamps after ischemia or congestion leads to a 15 min recovery phase to re-establish a stable baseline^53^.

Figure 4a–c shows a schematic illustration and pictures of the left rectus abdominus myocutaneous flap, including the artery and veins that control all blood exchange to the flap. Figure S13 presents pictures of a right rectus abdominus myocutaneous flap that highlights changes in color with different conditions at various phases of the experiment: baseline, ischemia, and congestion. Figure S13 also shows the vein and artery for blood exchange to this flap, as well as the ViOptix device. Figure 4d summarizes the results of local-tissue oxygenation determined from the implanted probe and the ViOptix device during two cycles of baseline, ischemia, recovery, congestion, and recovery. Additional measurements using the same probe on separate animals indicate good reproducibility (*n* = 3), as shown in Fig. S14. The tissue oxygenation drops upon ischemia or congestion, and returns to a normal range upon recovery. Although the absolute value of StO_2_ from intramuscular and skin-mounted measurements cannot be compared directly, the normalized cross-correlation analysis at zero lag is >0.99, indicating strong qualitative correspondence, as might be expected. Statistical analysis of in vivo StO_2_ measurement using Bland-Altman method, shows a mean difference of −0.30 ± 7.3% (mean ± SD, complete event phases, 130 min of continuous measurement, Fig. S15) when compared to commercially available skin-mounted ViOptix.

Figures 4e and S16 summarize long-term studies of the functionality of the NIRS probe and demonstrate its potential for long-term applications. The measurement from the fingertip after immersion in PBS at 37 °C for up to 4 weeks demonstrates that the encapsulation barrier prevents biofluid penetration and probe failure. As this measurement also depends on the interface between fingertip and probe, it only reflects the probe lifetime but does not determine the signal quality. Previous reports on other types of optical probes also demonstrate stable in vivo operation for at least 1 week and up to 2 months^33,54^. Another ongoing long-term study demonstrates stable operation for over one month. These results suggest potential applications for long-term StO_2_ monitoring.

### In vivo demonstration in a porcine kidney model

Demonstrations in a porcine kidney model illustrate potential applicability in monitoring the reconstruction and regrowth of organ transplants.

Here, the experiment proceeds with open surgery to raise the kidney first for clear illustration and visual comparison during the ischemia and congestion phases. Minimally invasive procedures to insert the probe into the kidney with a peel-away sheath introducer, as used with catheters or minimally invasive percutaneous nephrolithotomies, should be straightforward to implement (Fig. S12)^55^. The surgical processes for raising one kidney and implanting the devices appear in the Methods section. As in the muscle-flap model, in these experiments, the kidney undergoes 15 min cycles of baseline, ischemia with clamped artery, recovery after unclamping artery, congestion with clamped veins, and recovery phases.

Figure 5a–c shows schematic illustrations of a raised kidney with an implanted probe and the artery and vein for blood exchange. Figure S17 highlights visible color changes at various phases of the experiment: baseline, ischemia, and congestion. This figure also shows vessel loops for clamping the vein and artery. The results of local-tissue oxygenation measured in the kidney with the probe during two cycles of ischemia and congestion appear in Fig. 5d, with representative data in Fig. S18. Additional measurements from another independent device on the same animal (Fig. S19) illustrate the reproducibility of the results, and the correspondence between data collected from two independent probes (normalized cross-correlation at zero lag >0.999). Statistical analysis of measurements from two devices using Bland-Altman method, show a mean difference of −0.42 ± 6.2% (mean ± SD, complete event phases, 130 min of continuous measurement, Fig. S20). The data presented in Fig. S18b highlight the patterns of pulsation that result from blood flow inside the kidney. Fourier transforms of the raw data (Figs. 5e and S21) show changes in heartbeat signal (at 1.7–2.5 Hz) and respiration signal (at ~0.3 Hz) during the occlusion of blood vessels to flap and kidney. The strength of the pulsatile component of the signal decreases during ischemia/congestion, as a simple, binary indication of blood flow.

## Discussion

The results reported here integrate a collection of technology advances as the basis for an implantable probe that not only offers capabilities in continuous, real-time, wireless, monitoring of regional tissue oxygenation at targeted depth but also enhances its sensing stability and accuracy through differential and spatially resolved near-infrared spectroscopy. The system design follows explicitly from a consideration of critical needs in clinical settings, particularly for free muscle-flap transfers and organ transplantations. The small diameter of the probe minimizes invasiveness and potential adverse effects on the neighboring tissue, the dual-photodetector configuration enhances measurement accuracy and the bioresorbable barbs provide a self-anchoring mechanism that both eliminates the surgical steps of suturing and ensures a safe post-operation extraction. Biocompatibility studies show no measurable adverse effects from the device implantation to organs or surrounding tissue. Continuous monitoring of tissue oxygenation in muscle flaps and kidneys in live porcine models demonstrates feasibility in a muscle-only free flap and organ monitoring with high sensitivity, reliability, and reproducibility. Further clinical validation and testing may lead to broad adoption in both hospital settings and rehabilitation centers.

Compared to previous StO_2_ monitoring devices, the sub-mm form factor of this device enables minimally invasive use in deep tissue. The use of two μ -ILEDs and two μ -IPDs enables spatially resolved spectroscopy methods for measuring StO_2_ without assumed optical parameters. The probe can also capture SpO_2_ and HR as well. The focus of the present studies is to validate the performance of continuous monitoring of StO_2_ in deep tissues and organs. Various enhancements can be considered to facilitate applications within and beyond clinical settings. For example, wireless charging capabilities will allow implantation of the electronic module to eliminate risks of externalized hardware for long-term monitoring. In such scenarios, the influence of motion on measurement accuracy must be carefully considered. Other areas of opportunity are in the development of minimally invasive deployment procedures via peel-away sheath introducers.

## Methods

The Institutional Animal Care and Use Committee at Washington University, School of Medicine approved the protocol (21-0134) for animal studies.

### Design and fabrication of the wireless implantable near-infrared spectroscopy optoelectronic microsystem

The electronic module was designed in EAGLE 9.6 (Autodesk Inc.). The fabrication began with patterning a flexible PCB (thicknesses of constituent layers, Cu/PI/Cu, 18/75/18 μm) by laser ablation, to define the circuit interconnects, the bonding pads for the electronic components, and the geometry of the probe. Hot-air soldering with low-temperature soldering paste (Bi57Sn42Ag1, Chip Quik, Inc.) bonded the electronic components, μ-ILEDs (TCSD14-660 and TCSD14-850, III-V Materials, Inc.), and μ-IPDs (T1197P6, Vishay Semiconductors) to respective pads. The BLE microcontroller (NRF52832, Nordic Semiconductor Inc) was programmed using Segger Embedded Studio to define protocols for the μ-ILEDs illumination sequence, data sampling, and data communication. The GUI was developed using Python 3.9. The resistances of the feedback resistors for the transimpedance amplifiers were 1 MΩ and 4.3 MΩ for μ-IPD1 and μ-IPD2, respectively. After assembling the electronic components, chemical vapor deposition (Specialty Coating Systems, PDS2010 LABCOTER2 Parylene Deposition System) fully encapsulated the device with a layer of 14 μm-thick parylene as a biofluid barrier. Dip coating established an overcoat of silicone (Silbione RTV 4420) on the BLE module, using procedures described in a previous report^23,56^. The current design supports operational lifetimes set by the capacity of the battery. Future versions can incorporate wireless charging capabilities.

### Preparation of bioresorbable barbs

The process began with dissolving Poly(D, L-lactide-co-glycolide) (PLGA, lactide: glycolide (50:50), mol wt 30,000–6000, Sigma–Aldrich/Millipore Sigma) with ethyl acetate at a concentration of 5% w/v. Drop casting 8 mL of the PLGA solution onto a 4 inch Si wafer with a self-assembled silane monolayer (trichloro(1H,1H,2H,2H-perfluorooctyl) silane, Sigma–Aldrich/Millipore Sigma), evaporating the solvent in a fume hood overnight, then baking at 70 °C for 2 h yielded PLGA film with thicknesses of ~100 μm. A laser cutter (LPKF ProtoLaser R) defined the outline of the PLGA barbs that attached to the backside of the probe using a pressure by finger pressing and baking at 65–70 °C.

### Characterization of the electrical, optical, and thermal properties

The I–V characteristics of μ-ILEDs were measured with a probe station (Keysight B1500A Semiconductor Analyzer) to evaluate the power consumption.

Spectroscopic measurements with an integrating sphere (FOIS-1 Fiber Optic Integrating Sphere, Ocean Optics) and a spectrometer (FLAME-S-UV-VIS Miniature Spectrometer, Ocean Optics) yielded the emission spectra (absolute irradiance). Integration of the irradiance across relevant ranges of wavelengths generated the optical power at various currents (Keithley 6221 DC and AC current source, ATektronic Company). Recording the output voltage of the μ-IPDs yielded calibration curves for the intensity of red and NIR light separately.

An NTC thermistor (Murata Electronics), directly attached on top of the μ-ILEDs allowed measurements of changes in temperature. A digital multimeter (USB-4065, National Instruments), captured the changes in resistance during operation, to define the change in temperature according to ${{{{{\rm{R}}}}}}={{{{{{\rm{R}}}}}}}_{0}{{\exp }}{{{{{\rm{B}}}}}}(\frac{1}{{{{{{\rm{T}}}}}}}-\frac{1}{{{{{{{\rm{T}}}}}}}_{0}})$, ${{{{{{\rm{R}}}}}}}_{0}=10{{{{{\rm{k}}}}}}$, ${{{{{\rm{B}}}}}}=3380$.

### Optical simulation of the optoelectronic microsystems using Monte Carlo Method

Simulations of light propagation from the μ-ILEDs in biological tissues used Monte Carlo methods applied across a 3D computational space with 1000 × 1000 × 1000 bins of 5 × 10^−9^ cm^3^ volume and a total of 11 × 10^6^ photon packets for each simulation. μ-ILEDs with peak emission wavelengths at 660 nm (red) and 850 nm (NIR), respectively, served as light sources, with both active illumination areas of 0.25 × 0.25 mm and emission angles of ±60°. The irradiances at the illumination surfaces for both setups were 8.13 mW/mm^2^ for red and 12.1 mW/mm^2^ for NIR. Light propagation was simulated in muscular tissue with the corresponding wavelength-dependent scattering anisotropy factor (${{{{{{\rm{g}}}}}}}_{{{{{{\rm{Red}}}}}}}=0.87$, ${{{{{{\rm{g}}}}}}}_{{{{{{\rm{NIR}}}}}}}=0.93$), absorption coefficient (${{{{{{\rm{\mu }}}}}}}_{{{{{{\rm{a}}}}}}-{{{{{\rm{Red}}}}}}}=0.53\;{{{{{{\rm{cm}}}}}}}^{-1}$, ${{{{{{\rm{\mu }}}}}}}_{{{{{{\rm{a}}}}}}-{{{{{\rm{NIR}}}}}}}=0.49\;{{{{{{\rm{cm}}}}}}}^{-1}$) and reduced scattering coefficient (${{{{{{\rm{\mu }}}}}}}_{{{{{{\rm{s}}}}}}-{{{{{\rm{Red}}}}}}}^{{\prime} }=8.53\;{{{{{{\rm{cm}}}}}}}^{-1}$, ${{{{{{\rm{\mu }}}}}}}_{{{{{{\rm{s}}}}}}-{{{{{\rm{Red}}}}}}}^{{\prime} }=6.67\;{{{{{{\rm{cm}}}}}}}^{-1}$)^32,39,57^.

### Thermal simulation of the optoelectronic microsystems using finite element analysis

Analysis of heat transfer and dissipation used the commercial software COMSOL 5.5 (Heat Transfer Model) to quantify the change in temperature (∆*T*) in the surrounding muscular tissue as a result of the thermal power associated with the operation of the µ-ILEDs and absorption of light emitted from them. The simulation assumed an ideal constant background of tissue by neglecting heat generated from tissue metabolism and the effects of blood perfusion.

Thus, the Pennes’ bioheat equation^58–60^ is written as

4 $$\rho {Cp}\frac{\partial T}{\partial t}+\nabla \cdot \left(-k\nabla T\right)={Q}_{{{{{{{\mathrm{the}}}}}}}}+\phi \mu a$$

where *T* is the temperature; *t* is the time; *k*, ρ, and *C*_p_ are the thermal conductivity, mass density, and heat capacity of the tissue, respectively; and *Q*_the_ is the heat generated from the thermal power of the µ-ILEDs. The heat associated with light emission was calculated as the product of the light fluence rate ϕ obtained in the optical simulation and the absorption coefficient μ_a_ of the tissue^23^. The tissue, probe structure, and associated components were modeled using four-node tetrahedral elements. Convergence tests of the mesh size were performed to ensure accuracy. The total number of elements in the models was ~650,000.

### Measurement of signal intensity as a function of LED-PD distance

Assembling an NIR µ-ILED and a µ-IPD separately onto two respective PCB boards yielded measurements of signal intensity as a function of LED-PD distance as embedded inside a piece of pork belly. A DC power supply connected to the µ-ILED delivered a constant power (0.4 mW power, 2 V output voltage). A digital multimeter allowed continuous recording of the photocurrents measured from the µ-IPD at various separation distances. Plotting the averaged photocurrents and associated error bars based on the measurements yielded a comparison of signal intensity as a function of distance, as a basic design guideline.

### Mechanical characteristics of the bioresorbable barbs

Mechanical measurements using a tensile tester (Mark-10 ESM303 instrument) with a 10 N force gauge (Mark-10 Corporation) yielded profiles of pulling force for probes with or without barbed structures. The probes were immersed in PBS solution at 37 °C before the tests. At various times of immersion, the probes were removed and implanted into porcine meat with a surgical blade (size: #11). The motor of the Mark-10 traveled at a constant rate of 0.2 mm/s, measuring the distance and force. Each probe design was tested at least three times.

### In vitro oxygenation measurements to compare to a commercial probe

The oxygenation level of fresh defibrinated horse blood (100 mL, Fisher Scientific) was tuned by adding sodium dithionite (MilliporeSigma) to deoxygenate hemoglobin. Immersing a NIRS probe into the horse blood mixed with various concentrations of sodium dithionite yielded a collection of measurements at respective oxygenation levels. A standard, commercial blood gas analyzer (Abbott Point of Care i-SAT CG8+ Cartridge) measured the stabilized oxygenation level as reference.

### In vivo evaluation of biocompatibility

Female Sprague Dawley rats (6–8 weeks old) were purchased from Charles River Laboratories (O Fallon, MO). All animal handling and experimental protocols were approved by the Institutional Animal Care and Use Committee at Northwestern University. Rats were housed three per cage and maintained on a 12:12 light:dark cycle and provided ad libitum access to food and water throughout the study. The NIRS sensing probes with or without bioresorbable PLGA barbs were sterilized by autoclave, where the probe was about 2.5–3 cm including all the components and barbs. On the surgery day (Day 0), the rats (185–205 g) were anesthetized using isoflurane (2.5% in O_2_, to effect), and an incision (∼1.0 cm in length) perpendicular to the spine was made between the shoulder blades using a scalpel blade. To house the device, a small pocket was formed under the skin using blunt scissors. The devices were placed in the pocket and the incision was closed with wound clips. Rats in the control group underwent sham surgery in which both the incision and pocket were made, but no device was implanted. All rats were given analgesics pre- and post-surgery as required per IACUC protocol. Following surgery, rats were monitored daily for general health conditions and behavior. The body weights for each rat were also recorded twice per week.

To evaluate the device immune-toxicity effect, blood samples were collected from all animals at 1-, 4-, and 6-weeks post-implantation. Blood samples were collected using a restrainer and in an awake rat via tail vein puncture. For hematology analysis, a 200–250 μL of blood volume was collected in K_2_-EDTA tubes. For serum clinical blood chemistry analysis, 500–600 uL of blood was collected in uncoated tubes. Blood sample analysis was performed by Veterinary Diagnostic Laboratory at University of Illinois.

At study termination, major organs including heart, lung, liver, kidney, spleen, and brain in addition to skin adjacent to the implantation site were collected. Organs were then weighed and placed in 10% buffered formalin fixative. For histopathology studies, tissue samples were embedded in paraffin, sectioned, and stained using hematoxylin and eosin (H&E). Tissue morphology was evaluated to identify fibrosis (40× fields) and mixed-cell inflammation and hemorrhage (400× fields) from ten distinct regions per sample.

### Micro-CT imaging

For tests of biocompatibility, rats with probes implanted subdermally in the dorsal interscapular region were anesthetized in an induction chamber with ~2.5% isoflurane in O_2_ and transferred to the imaging bed with continuous isoflurane delivery via nose cone at 1 to 2% at 1 day, 5 days, 7 days, 2 weeks, 4 weeks, and 6-weeks post-implantation for micro-CT imaging. Images were acquired using a Mediso NanoScan PET/CT imaging system with the following parameters: ‘medium’ magnification, 33 μm focal spot, 1 × 4 binning, with 720 projection views over a full circle, 300 ms exposure time, and 70 kVp (where kVp is peak voltage). The projection data were reconstructed with a voxel size of 68 μm using filtered (Butterworth filter) back-projection software from Mediso Nucline (v2.01). Reconstructed images were filtered with Amira v2020.3 (Thermo-Fisher) using a non-local means filter. Filtered data was segmented in Amira to highlight the device and 3D surface renderings were created using different colormaps for the skeleton and the device.

To quantify device movement, all skeleton datasets were registered to the first timepoint, and the resulting transformations were applied to the corresponding devices. The transformed devices were then resampled onto a single grid. The device labels were converted into surfaces, and the ‘align surfaces’ module was used to calculate the transformation between each consecutive pair of timepoints. The final translation measurement T was computed as the magnitude of the translation vector $< {x}_{T},{y}_{T},{z}_{T} >$: T = $\sqrt{{{x}_{T}}^{2}+{{y}_{T}}^{2}+{{z}_{T}}^{2}}$.

### In vivo experiments using porcine flap and kidney model

The Institutional Animal Care and Use Committee at Washington University, School of Medicine approved the protocol (21–0134). Six live pigs were utilized in different experiments (Landrace cross male pig, 3–4 months). Telazol, ketamine, and xylazine anesthetized the animals, followed by maintenance with inhaled isoflurane. After completion of all experiments, the animals were euthanized with pentobarbital.

### Rectus abdominus myocutaneous flap ischemia and congestion model

The flap harvest procedures were adapted from ref. ^53^. Both left and right pedicled rectus abdominus myocutaneous flaps were raised with the superficial superior epigastric vein, and the deep superior epigastric artery and veins separated for occlusion. A #15 blade was utilized to make an incision along muscle fibers near the bottom surface of the rectus abdominus muscle. The probe was inserted into the muscle with the bioresorbable barbs securing its position after insertion. A ViOptix device was mounted onto the skin paddle and connected to an external monitor. After 15 min of stable baseline, an Acland clamp applied to the right deep superior epigastric artery to induce complete ischemia and maintained for 15 min. Then the Acland clamp was released and re-established the stable baseline for 15 min. Acland clamps applied to both deep and superficial superior epigastric veins induced venous congestion and maintained for 15 min. Then the Acland clamps were released and allowed the flap to recover for 15 min. The entire cycle was repeated once.

### Kidney ischemia and congestion model

The peritoneal cavity was accessed through a laparotomy incision. The small bowel was retracted, and the posterior peritoneum was incised over the left kidney and renal vessels. The kidney was separated from all surrounding tissues bluntly, and isolated upon the renal vein, renal artery, and the ureter. Vessel loops were double-looped around the artery and vein to allow intermittent and atraumatic occlusion. The sensor was deployed within the kidney by puncturing its capsule with a #11 blade. The probe was advanced bluntly through this capsular rent, where it was quite stable. After the establishment of a flow baseline, renal artery thrombosis was simulated by placing tension on the vessel loop encircling the artery. Ischemia was confirmed by the obvious pallor of the organ. After 15 min, the vessel loop was loosened, and perfusion was re-established. After 15 min, a similar procedure was used to simulate renal vein thrombosis, and congestion was confirmed by observing purple discoloration of the organ. After 15 min venous occlusion was released, and reperfusion was allowed.

### Calculation of StO_2_ via spatially resolved spectroscopy

With the two photodetectors placed at different distances from the light sources, the spatial derivative of the optical density, $\frac{\partial A}{\partial \rho }$, can be estimated, where *ρ* is the distance from the origin, *I*_0_ and *I*(*ρ*) are the light intensities at the origin, and the distance *ρ*, *A* is the optical density, also called absorbance, defined as:

5 $$A\left(\rho \right)=-{{{\log }}}_{10}\left(\frac{I\left(\rho \right)}{{I}_{0}}\right)$$

Spatially resolved spectroscopy (SRS) at two different wavelengths in combination with the radiation transfer equation enabled a calculation of tissue oxygenation saturation level as in Eq. (2)^61^.

With the approximation ${\mu }_{a}\ll {\mu }_{s}^{{\prime} }$ and the assumption of a semi-infinite geometry, the SRS yields:

6 $$\frac{\partial A\left(\lambda \right)}{\partial \rho }=\frac{1}{{{{{{\rm{ln}}}}}}\left(10\right)}\left(\sqrt{3{\mu }_{a}{\mu }_{s}^{{\prime} }}+\frac{2}{\rho }\right)$$

Where *μ*_*a*_ is the absorption coefficient and ${\mu }_{s}^{{\prime} }$ is the reduced scattering coefficient, and ${\mu }_{s}^{{\prime} }$ usually can be modeled in the NIR range as:

7 $${\mu }_{s}^{{\prime} }=k\left(1-h\lambda \right)$$

Where *k* is a constant and *h* is the normalized slope between the reduced scattering coefficient and wavelength, dependent on tissue types, and is assumed to be invariant between individuals. Combining Eqs. (6) and (7) generates:

8 $$k{\mu }_{a}=\frac{1}{3\left(1-h\lambda \right)}{\left({{{{{\rm{ln}}}}}}\left(10\right)\frac{\partial A\left(\lambda \right)}{\partial \rho }-\frac{2}{\rho }\right)}^{2}$$

The absorption coefficient can also be estimated as below:

9 $${\mu }_{a,{\lambda }_{j}}={{{{{\rm{ln}}}}}}\left(10\right){\varepsilon }_{{Hb},{\lambda }_{j}}{C}_{{Hb}}+{{{{{\rm{ln}}}}}}\left(10\right){\varepsilon }_{{Hb{{{{{\mathrm{O}}}}}2}},{\lambda }_{j}}{C}_{{Hb{{{{{\mathrm{O}}}}}2}}}$$

For a two-wavelength model, the equation can be written in the matrix as below:

10 $$\left[\begin{array}{c}k{\mu }_{a,{\lambda }_{1}}\\ k{\mu }_{a,{\lambda }_{2}}\end{array}\right]={{{{{\rm{ln}}}}}}\left(10\right)\left[\begin{array}{cc}{\varepsilon }_{{Hb},{\lambda }_{1}} & {\varepsilon }_{{Hb{{{{{\mathrm{O}}}}}2}},{\lambda }_{1}}\\ {\varepsilon }_{{Hb},{\lambda }_{2}} & {\varepsilon }_{{Hb{{{{{\mathrm{O}}}}}2}},{\lambda }_{2}}\end{array}\right]\left[\begin{array}{c}k{C}_{{Hb}}\\ k{C}_{{Hb{{{{{\mathrm{O}}}}}2}}}\end{array}\right]$$

which is equivalent to Eq. (1).

11 $$\left[\begin{array}{c}k{C}_{{Hb}}\\ k{C}_{{Hb{{{{{\mathrm{O}}}}}2}}}\end{array}\right]=\frac{1}{{{{{{\rm{ln}}}}}}\left(10\right)}{\left[\begin{array}{cc}{\varepsilon }_{{Hb},{\lambda }_{1}} & {\varepsilon }_{{Hb{{{{{\mathrm{O}}}}}2}},{\lambda }_{1}}\\ {\varepsilon }_{{Hb},{\lambda }_{2}} & {\varepsilon }_{{Hb{{{{{\mathrm{O}}}}}2}},{\lambda }_{2}}\end{array}\right]}^{-1}\left[\begin{array}{c}k{\mu }_{a,{\lambda }_{1}}\\ k{\mu }_{a,{\lambda }_{2}}\end{array}\right]$$

Therefore, tissue oxygen saturation can be calculated with Eq. (2).

12 $${St}{O}_{2}=\frac{{C}_{{Hb{{{{{\mathrm{O}}}}}2}}}}{{C}_{{Hb{{{{{\mathrm{O}}}}}2}}}+{C}_{{Hb}}}=\frac{{{kC}}_{{Hb{{{{{\mathrm{O}}}}}2}}}}{{{kC}}_{{Hb{{{{{\mathrm{O}}}}}2}}}+k{C}_{{Hb}}}$$

The following parameters were used for data analysis:^32,62^ ${\varepsilon }_{{Hb}{O}_{2},850\;{{{{{{\mathrm{nm}}}}}}}}=0.25$, ${\varepsilon }_{{Hb},850\;{{{{{{\mathrm{nm}}}}}}}}=0.19$, ${\varepsilon }_{{Hb}{O}_{2},660\;{{{{{{\mathrm{nm}}}}}}}}=0.08$, ${\varepsilon }_{{Hb},850\;{{{{{{\mathrm{nm}}}}}}}}=0.81$ (unit: ${{{{{\rm{L}}}}}}.{{{{{\rm{mmo}}}}}}{{{{{{\rm{l}}}}}}}^{-1}.{{{{{\rm{c}}}}}}{{{{{{\rm{m}}}}}}}^{-1}$), and *h* values between 5.3–6.5 × 10^−4^ nm^−1^ for 850 nm and 6.3–7.5 × 10^−4^ nm^−1^ for 660 nm.

### Data analysis

Data analysis was conducted with MATLAB (R2020b, MathWorks Inc.). Raw data first passed through a low-pass filter (0.2 Hz, second-order Butterworth digital filter) to extract the non-pulsatile (DC) component, and an average moving window of 5 s was applied when calculating the optical density and further calculation of StO_2_ as discussed above^30^.

### Statistics and reproducibility

Results are reported as mean ± SEM unless noted. No statistical method was used to predetermine sample size and no data were excluded from the analysis. The experiments were not randomized and the investigators were not blinded to allocation during experiments and outcome assessment.

### Reporting summary

Further information on research design is available in the Nature Research Reporting Summary linked to this article.

## Supplementary information

Supplementary Information

Reporting Summary

## Data Availability

All relevant data supporting the key findings of this study are available within the article and its Supplementary Information files, or from the corresponding authors upon reasonable request. Source data are provided with this paper.

## Source data

Source data
