# Supplementary material for: Wireless implantable optical probe for continuous monitoring of oxygen saturation in flaps and organ grafts
Source: Nat Commun. 2022 May 30;13:3009. doi: 10.1038/s41467-022-30594-z (PMC9151749; doi:10.1038/s41467-022-30594-z)
Supplement: Supplementary file 1 — Supplementary Information [file 41467_2022_30594_MOESM1_ESM.pdf]

Wireless Implantable Optical Probe for Continuous Monitoring of Oxygen Saturation in Flaps and Organ Grafts

Hexia Guo,[1,2†] Wubin Bai,[1,2,3†*] Wei Ouyang,[2†] Yihan Liu,[3] Changsheng Wu,[2] Yameng Xu,[4] Yang Weng,[1] Hao Zang,[1] Yiming Liu,[2] Lauren Jacobson,[5] Ziying Hu,[2] Yihang Wang,[3] Hany M. Arafa,[2] Quansan Yang,[2,6] Di Lu,[2] Shuo Li,[2] Lin Zhang,[3] Xun Xiao,[3] Abraham Vázquez-Guardado,[2] Joanna Ciatti,[2] Elizabeth Dempsey,[7] Nayereh Ghoreishi-Haack,[7] Emily A. Waters,[8] Chad R. Haney,[8] Amanda M. Westman,[5] Matthew R. MacEwan,[9] Mitchell A. Pet,[5*] John A. Rogers [1,2,6,10-12*]

[1]Department of Materials Science and Engineering, Northwestern University, Evanston, IL 60208, USA

[2]Querrey Simpson Institute for Bioelectronics, Northwestern University, Evanston, IL 60208, USA

[3]Department of Applied Physical Sciences, University of North Carolina at Chapel Hill, Chapel Hill, NC 27514, USA

[4]The Institute of Materials Science and Engineering, Washington University in St. Louis, St. Louis, MO 63110, USA

[5]Division of Plastic and Reconstructive Surgery, Department of Surgery, Washington University School of Medicine, St. Louis, MO 63110, USA

[6]Department of Mechanical Engineering, Northwestern University, Evanston, IL 60208, USA

[7]Developmental Therapeutics Core, Northwestern University, Evanston, IL 60208, USA

[8]Center for Advanced Molecular Imaging, Northwestern University, Evanston, IL 60208, USA

[9]Department of Neurosurgery, Washington University School of Medicine, St. Louis, MO 63110, USA

[10]Department of Biomedical Engineering, Northwestern University, Evanston, IL 60208, USA

[11]Department of Chemistry, Northwestern University, Evanston, IL 60208, USA

[12]Department of Neurological Surgery, Feinberg School of Medicine, Northwestern University, Evanston, IL 60208, USA

[†]These authors contributed equally to this work.

*To whom correspondence should be addressed. E-mail: wbai@unc.edu; pet@wustl.edu; jrogers@northwestern.edu .

Supplementary Information

Supplementary Note 1-2

Supplementary Figure S1-S21

Supplementary Note 1:

Study of biocompatibility of the implantable probe with bioresorbable barbs

Study of blood chemistry and average blood count through a 6-week period shows no evidence of toxicity of the NIRS probe. Blood chemistry results show no significant difference from control values (Figure 2i and Figure S6a), indicating normal enzyme and electrolyte levels, consistent with absence of organ-specific diseases or metabolic disorders. Specifically, normal levels of aminotransferase, alkaline phosphatase, aspartate transaminase, albumin, alanine, and total protein indicate normal liver function.[1] Normal levels of blood urea nitrogen and creatinine suggest normal kidney function.[2] Normal levels of sodium, potassium, calcium, chloride, phosphorus, and glucose suggest normal functions of the metabolic system.[3] Average counts of red blood cells, white blood cells, hematocrit, hemoglobin, mean corpuscular hemoglobin, mean corpuscular volume, and platelets all indicate no abnormalities, such as bleeding disorders, anemia, liver disease, heart attack or nutritional deficiency (Figure 2h and Figure S6b).

In Figure 2h and Figure S6c, histopathological analysis of major organs including brain, heart, lung, liver, kidney, and spleen indicates no noticeable increase in the count of immune cells caused by implantation of probes with and without bioresorbable barbs.

Supplementary Note 2:

Design considerations of the bioresorbable barbs

Figure S11 show the measured forces required for enable pulling or injecting motions, for various dimensional parameters, d and α, (as defined in Figure S11) respectively. The results indicate that increasing d (from 0.3 mm to 0.65 mm) and α (from 20° to 60°) increases both the pulling force and injecting force, while the increasing rate for the pulling force is larger than that of injecting force.

Pulling out an implanted probes with barbs from various organs (including heart, kidney, small intestine, stomach, and liver, Figure S10), and measuring the weight of tissue fragments dragged out by the barbs (Figure S11a) enable an estimation of damages resulted from the bioresorbable barbs during extraction of the NIRS probe right after the implantation. The experimental results indicate the pulling movement of the injected probe may induce damage of tissue with small amount at the order of milligram (Figure S11). Figure S11c&d show the measured forces required for enable pulling or injecting motions, for various dimensional parameters, d and α, (as defined in Figure S11) respectively. The results indicate that increasing d (from 0.3 mm to 0.65 mm) and α (from 20° to 60°) increases both the pulling force and injecting force, while the increasing rate for the pulling force is larger than that of injecting force.

Supplementary Figures

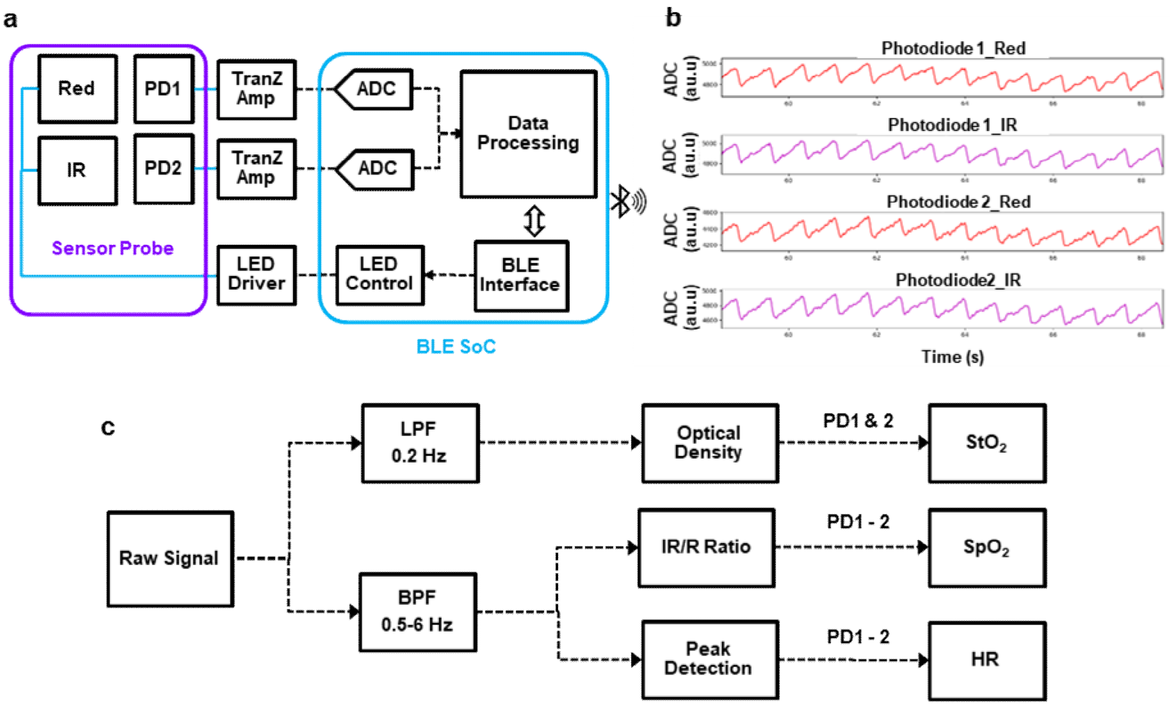

Figure S1. Data collection and analysis associated with operation of the wireless spectroscopic NIRS probing system. a) Schematic block diagram of the NIRS probe and Bluetooth Low Energy (BLE) module. b) GUI showing representative measurement results of pulse signals collected from the index finger. c) Algorithm flow chart of data analysis to extract tissue oxygenation saturation (StO_2), pulse oxygenation saturation (SpO_2), and heart rate (HR). LPF: low-pass filter, BPF: band-pass filter, IR: infrared, R: red.

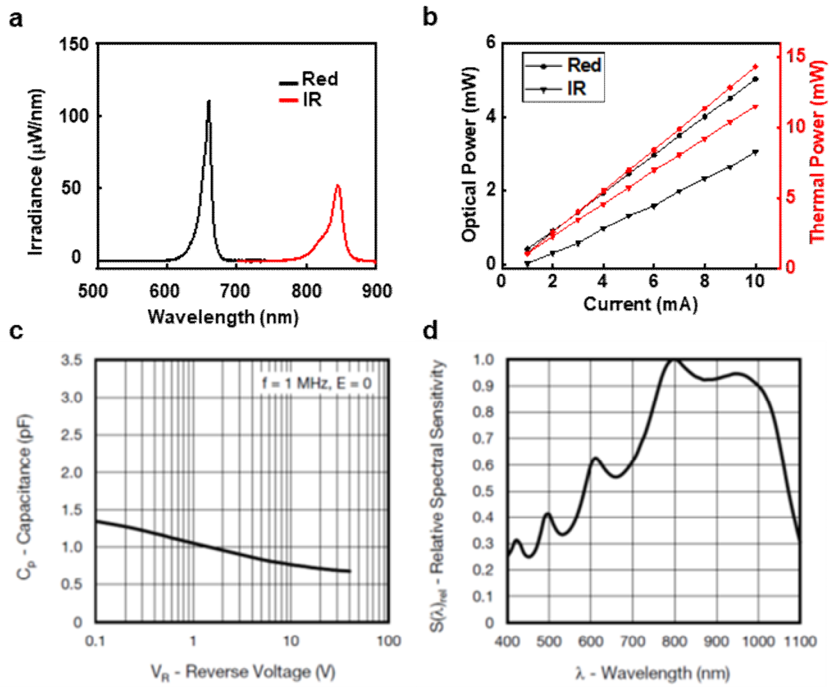

Figure S2. Characteristics of the μ-ILEDs and μ-IPD. a) Emission spectra of the μ-ILEDs. b) Optical and thermal power as a function of applied current. c) Measurement of diode capacitance of the μ-PD as a function of reverse voltage. d) Measurement of relative spectral sensitivity of the μ-PD as a function of wavelength without epoxy coating. Source data are provided as a Source Data file. c)&d) are cited from the device data sheet.

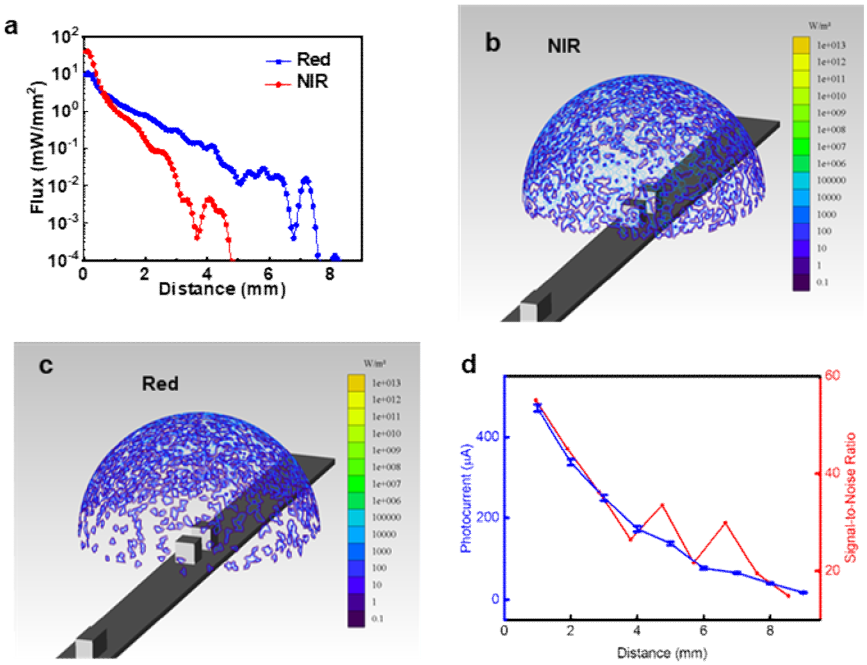

Figure S3. Optical simulations and experimental results. a) Flux distribution along the probe direction. b-c) Illumination volume associated with the NIR and red μ-ILEDs, respectively. d) Left: Measured photocurrent of a photodiode in response to illumination of a LED at various distances inside a porcine flap. Right: Calculated signal-to-noise ratio (SNR). Source data are provided as a Source Data file.

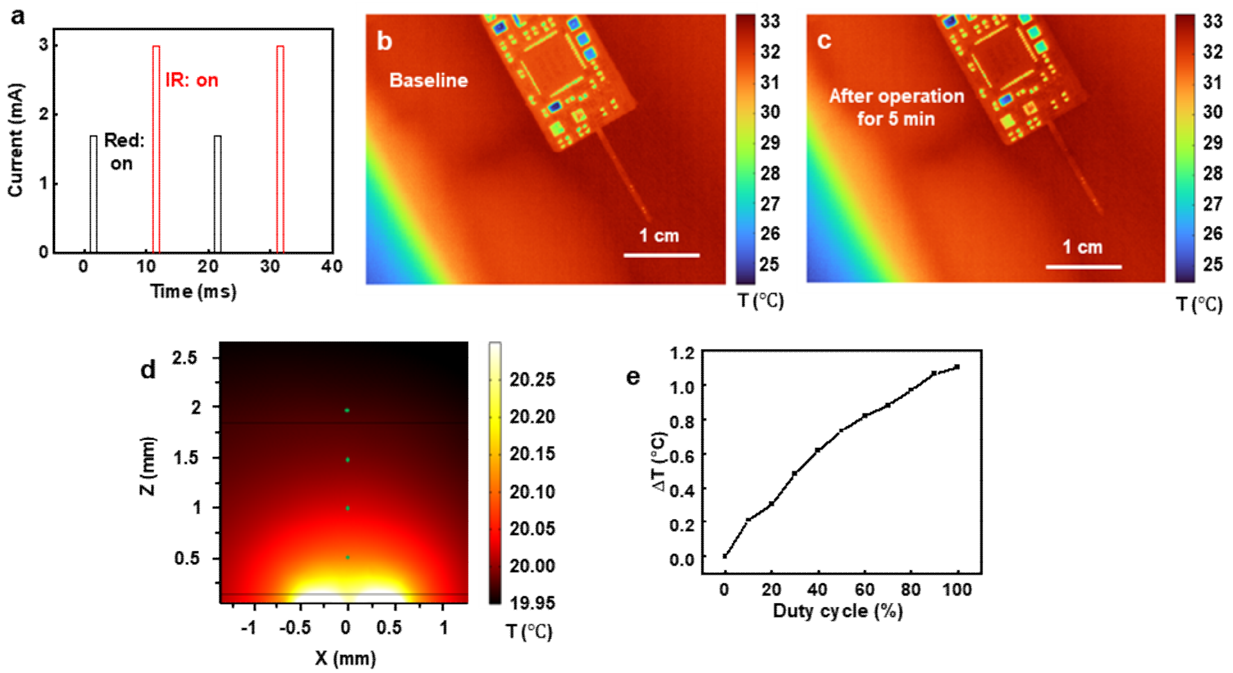

Figure S4. Electrical and thermal characteristics. a) Time dependent profiles of current supplied to the µ-ILEDs through the BLE microcontroller. b-c) Thermal images of the system resting on the forearm b) before activation and c) after operation for 5 min. d) Simulation results for the temperature distribution in the XZ plane at the center of the red and IR µ-ILEDs (Y=0 mm). e) Measured steady state temperature increase in porcine meat after 5 min at various duty cycle of µ-ILEDs. Source data are provided as a Source Data file.

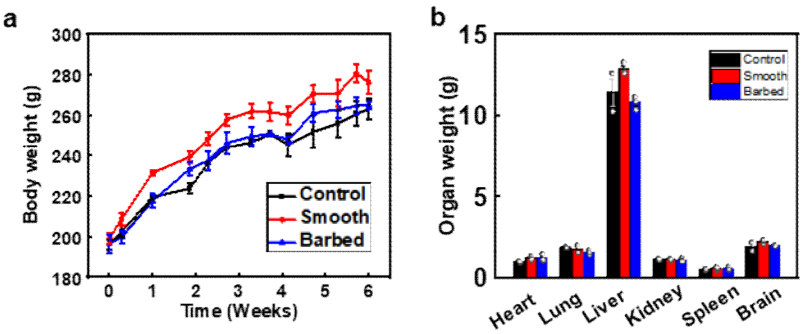

Figure S5. In vivo evaluations of the biocompatibility of the smooth and barbed NIRS probes in rat models. a) Changes of body weight of rats in each group (control, smooth, and barbed group, n=3). b) Comparison of organ weight of rats in each group at six weeks post-implantation (n=3). All data are shown as mean ± SEM. Source data are provided as a Source Data file.

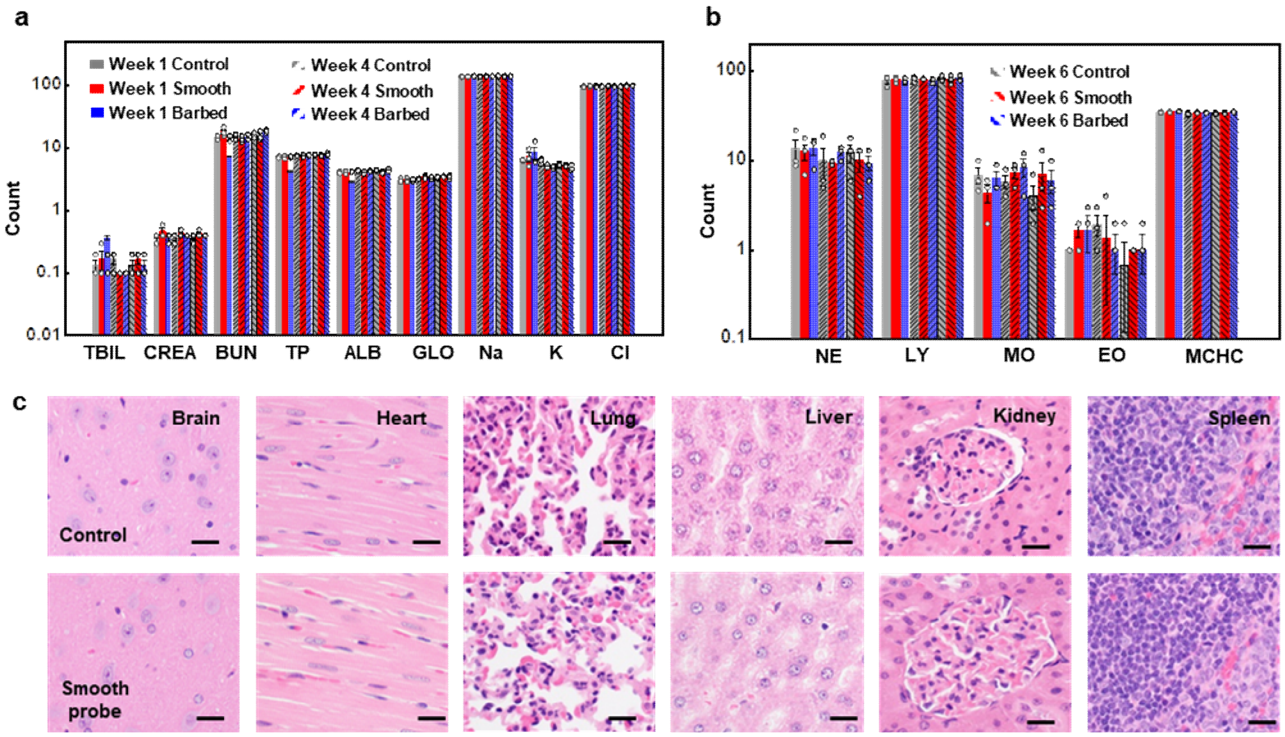

Figure S6. Biocompatibility of the implantable NIRS probe with bioresorbable barbs. Blood chemistry and blood counts tests for rats in a-b). TBIL: total bilirubin level (mg/dL), CREA: creatinine (mg/dL); BUN: blood urea nitrogen (mg/dL); TP: total protein (g/dL); ALB: albumin (g/dL); GLO: Globulin (g/dL); Na: sodium (mmol/L); K: potassium (mmol/L); Cl: chloride (mmol/L); NE: percentage of neutrophils (%); LY: percentage of lymphocytes (%); MO: percentage of monocytes (%); EO: percentage of eosinophils (%); MCHC: mean corpuscular hemoglobin concentration (g/dL). n=3 independent samples. All data are shown as mean ± SEM. c) Sections of brain, heart, lung, liver, kidney and spleen from rats in control (top row; left to right) and smooth implant (bottom row; left to right) groups. H&E, 400x. Scale bar: 25 um. n=3 independent samples with similar results. Source data are provided as a Source Data file.

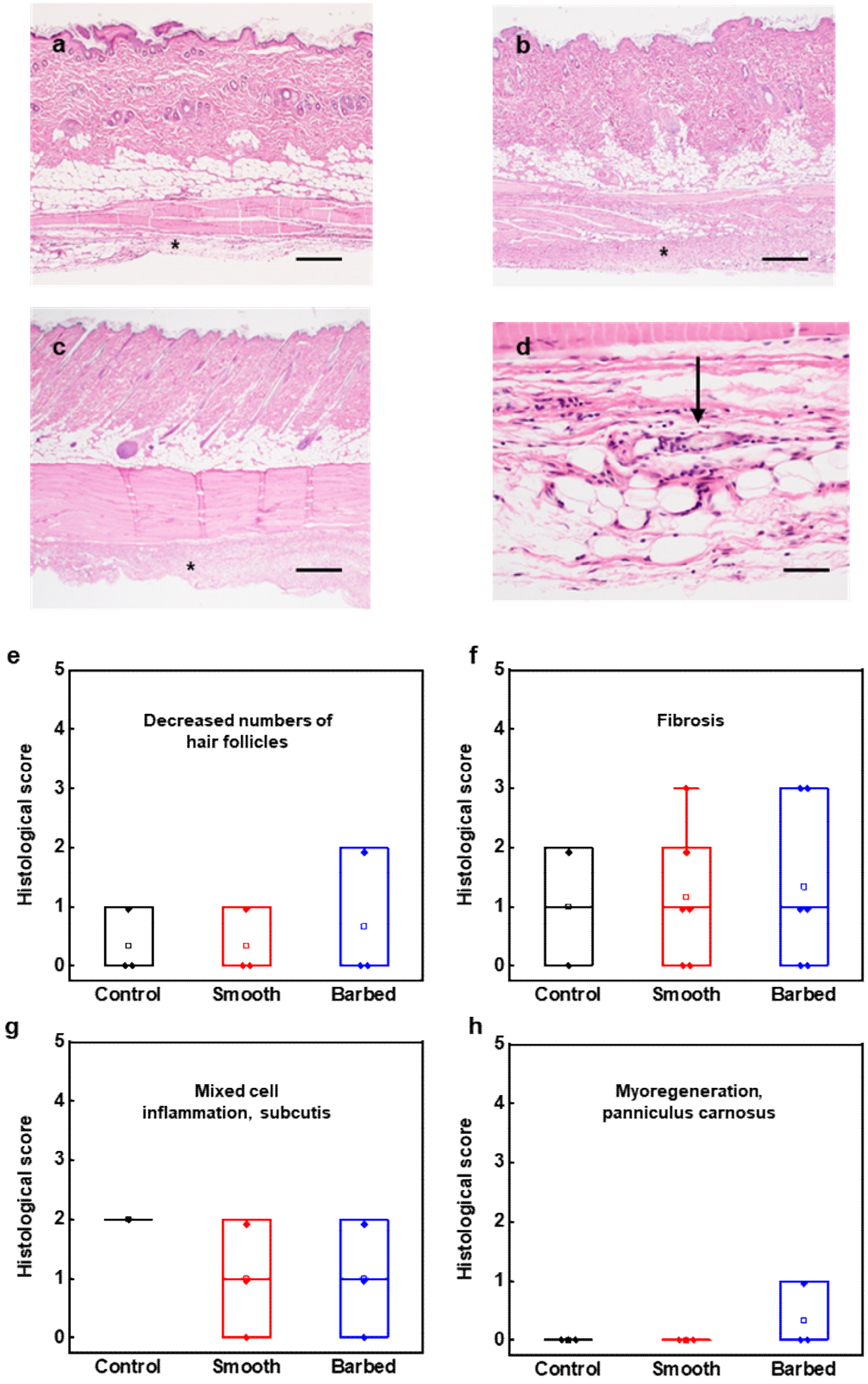

Figure S7. Histology studies of the implantable NIRS probe with bioresorbable barbs. Skin from implantation sites for control a), smooth b) and barbed c) groups. Subcutaneous fibrosis beneath the panniculus carnosus muscle (asterisks), hematoxylin and eosin (H&E), 40x. Scale

bar: 50 um. n=3 independent samples with similar results.Skin from a control rat. d) showing subcutaneous mixed-cell inflammation around an embedded hair shaft (arrow), H&E, 200x. e-f) Evaluation of histological score of skin tissue, where range 0-5 corresponds to histologically unremarkable, minimal, mild, moderate, marked, severe. n=3 independent samples. The boxplots show the median (center line), the first and third quartiles (lower and upper edge of the box, respectively), and the largest and smallest value that is ≤1.5 times the interquartile range (the limits of the upper and lower whiskers, respectively). Source data are provided as a Source Data file.

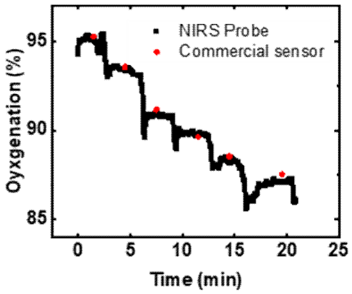

Figure S8. Oxygenation measured from both the NIRS probe, and a commercial sensor (Abbott i-SAT blood gas analyzer) immersed in horse blood at various ratios of HbO_2 and Hb. Source data are provided as a Source Data file.

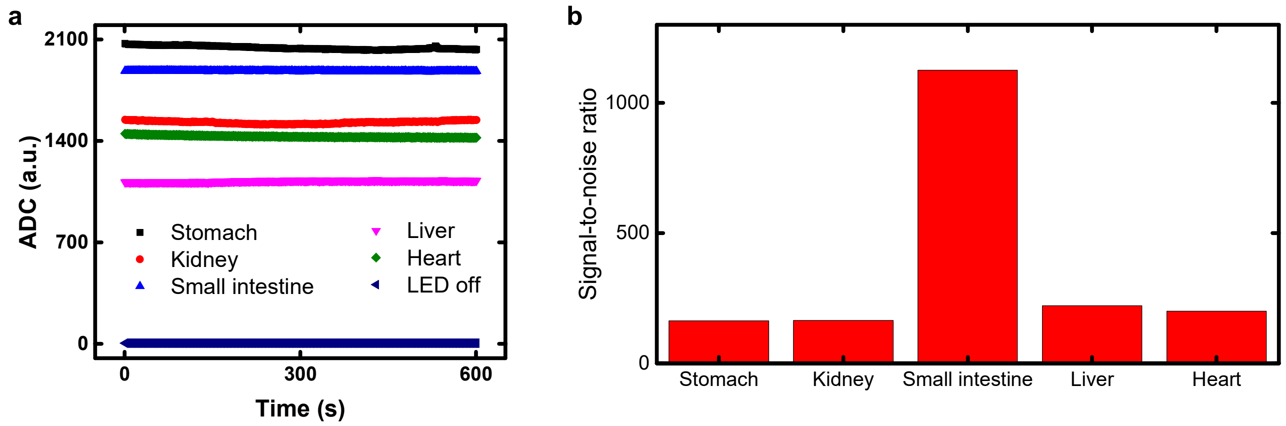

Figure S9. a) Measured signal intensities of a NIRS probe as its deployment inside various porcine organs, including stomach, kidney, small intestine, liver, heart, respectively. The effectively signal is significant compared with that of the probe with LEDs turned off. b) Calculated signal-to-noise ratio (SNR) of the NIRS probe as its deployment inside stomach, kidney, small intestine, liver, heart, respectively. Source data are provided as a Source Data file.

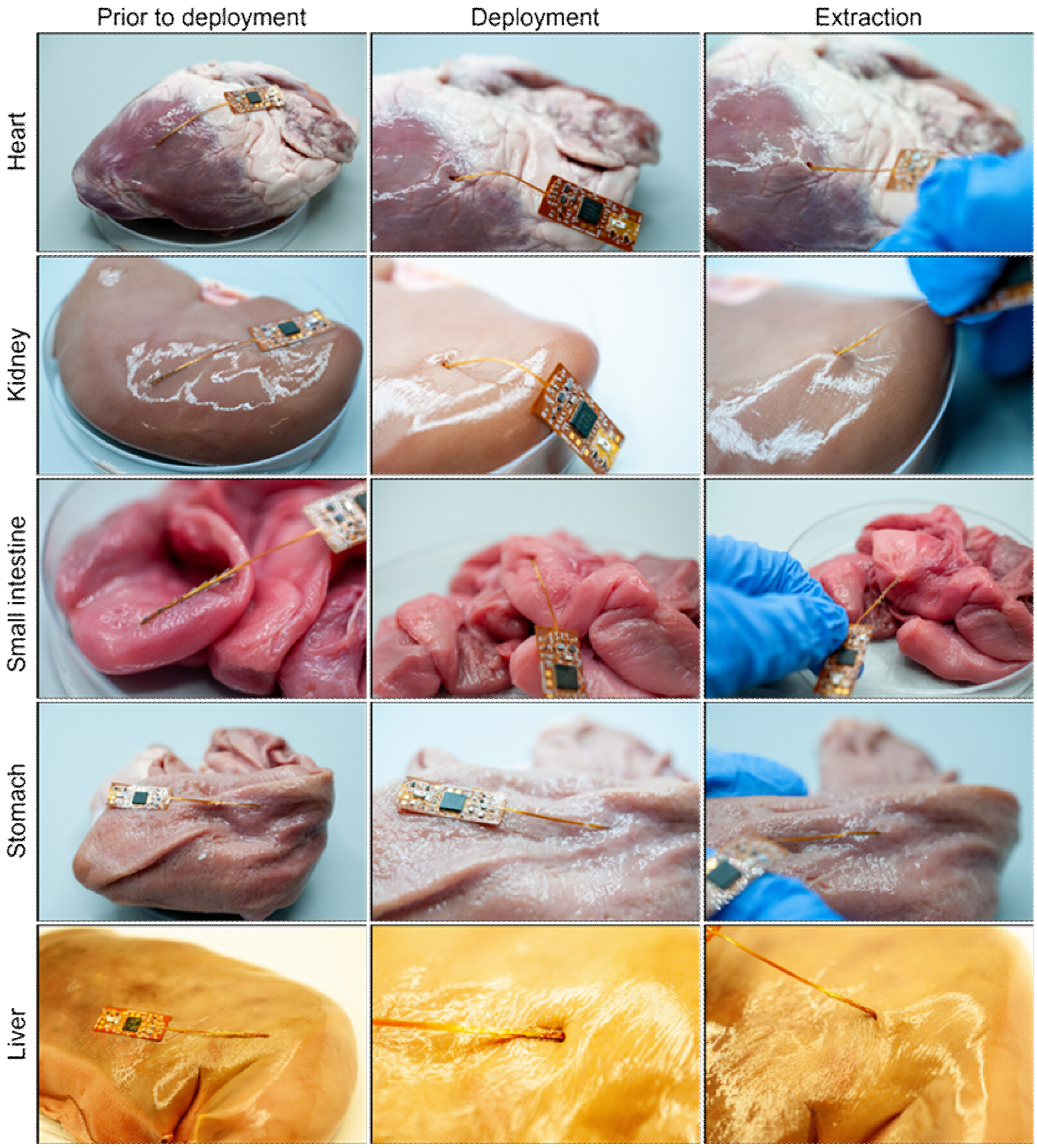

Figure S10. Images of a NIRS probe in the process of deployment into and extraction out of various organs, including heart, kidney, small intestine, stomach, and liver.

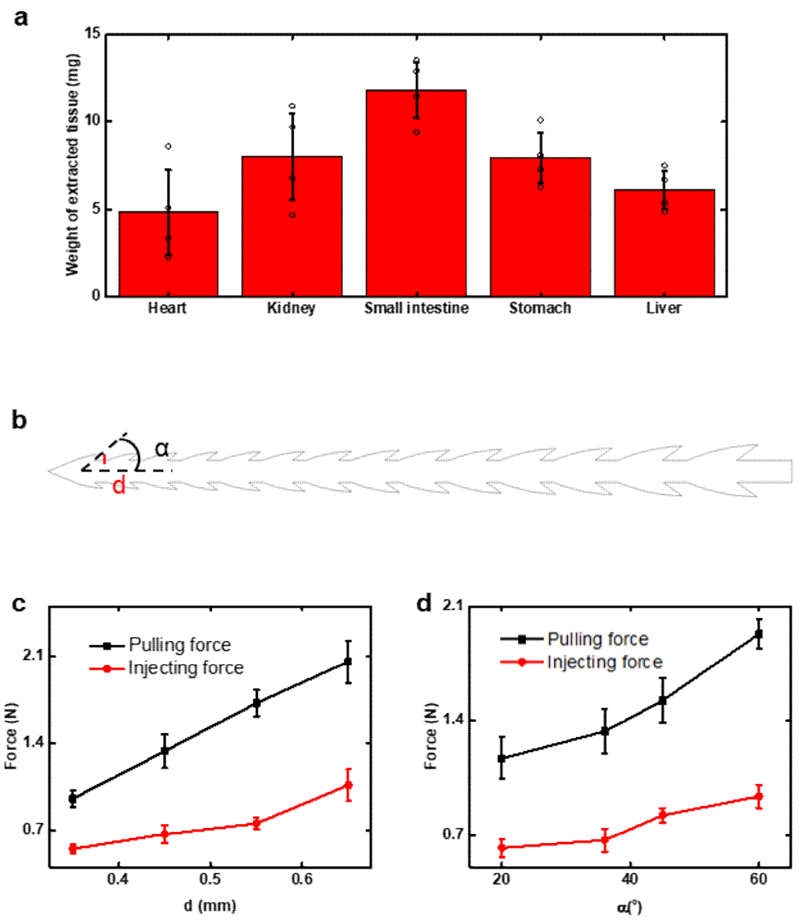

Figure S11. a) Measured weight of tissue fragments dragged out by the movement of pulling out a sensor probe with barbs implanted into various organs at a fixed depth (2 cm) and pulling speed (5mm/s). The design parameters of the barbs used here are α=22°, d=245 μm. b) schematic illustration showing the 2D layout of the bioresorbable barbs. Dimensional parameters, labelled as α and d, define the design characteristics of the barbs. c) Measured force (pulling and injecting) of NIRS probes integrated with barbs as a function of the dimensional parameter, *d*. d) Measured force (pulling and injecting) of NIRS probes integrated with barbs as a function of the dimensional parameter, *α*. All data are shown as mean ± SEM. Source data are provided as a Source Data file.

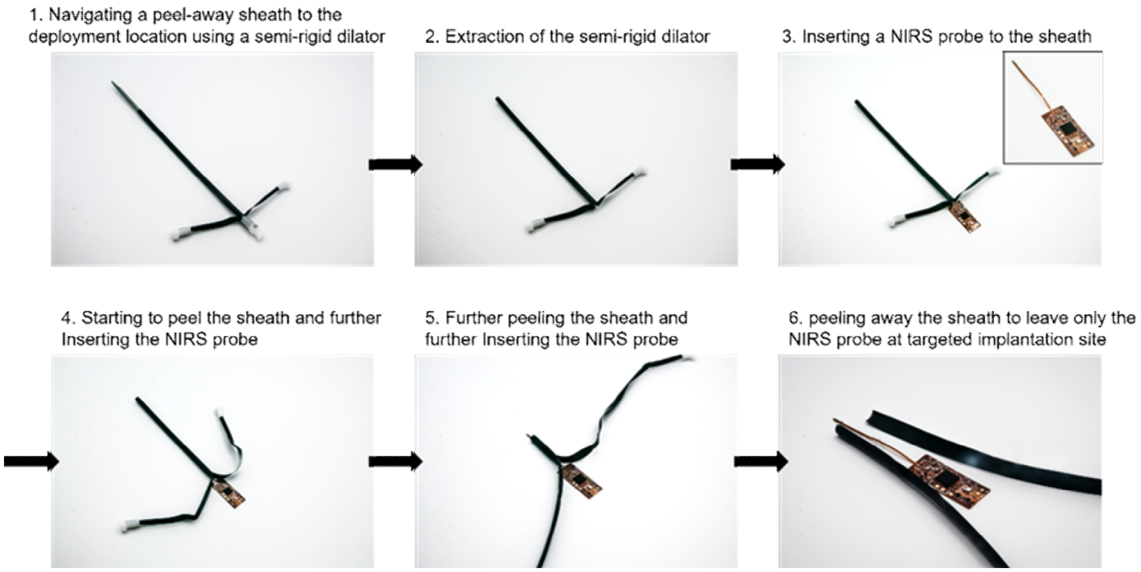

Figure S12. Demonstration of an approach to deploying a NIRS probe with a peel-away sheath. This scheme ensures that the anchoring effect of the bioresorbable barbed structure engages only after final deployment.

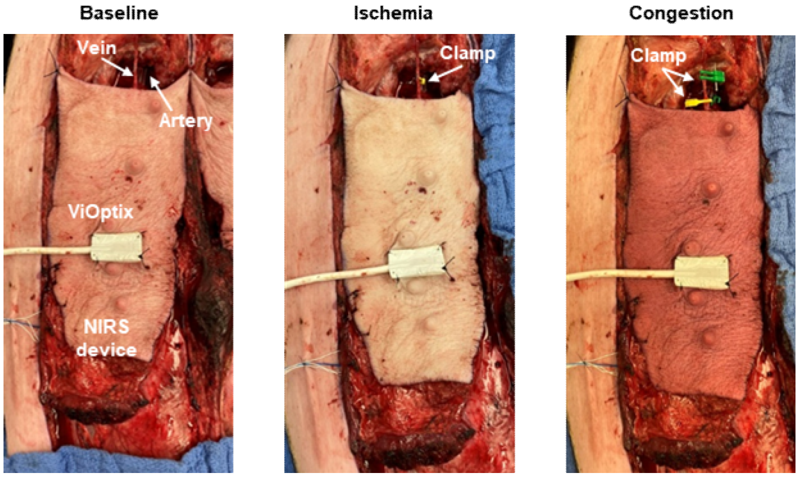

Figure S13. Studies using a NIRS probe in a porcine flap model. Images of the NIRS probe implanted in the right rectus abdominus myocutaneous flap and the ViOptix device attached on its skin paddle for various conditions: baseline, ischemia (artery clamped), and congestion (veins clamped) from left to right.

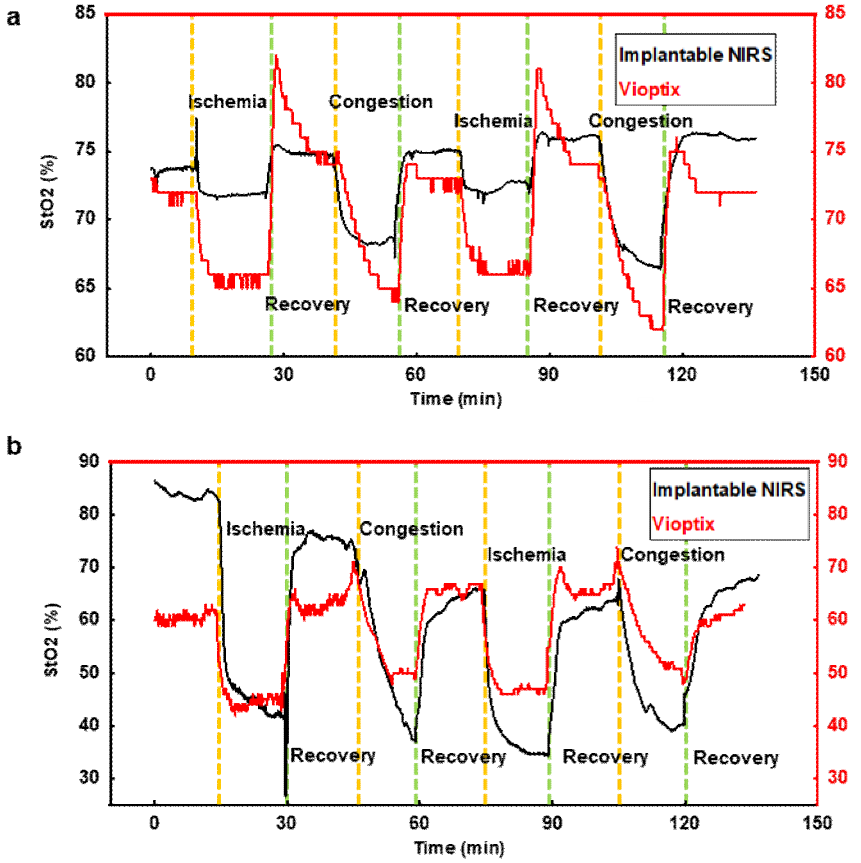

Figure S14. Additional StO_2 measurements from the NIRS probe in porcine flap model : a) Animal #2, b) Animal #3. Source data are provided as a Source Data file.

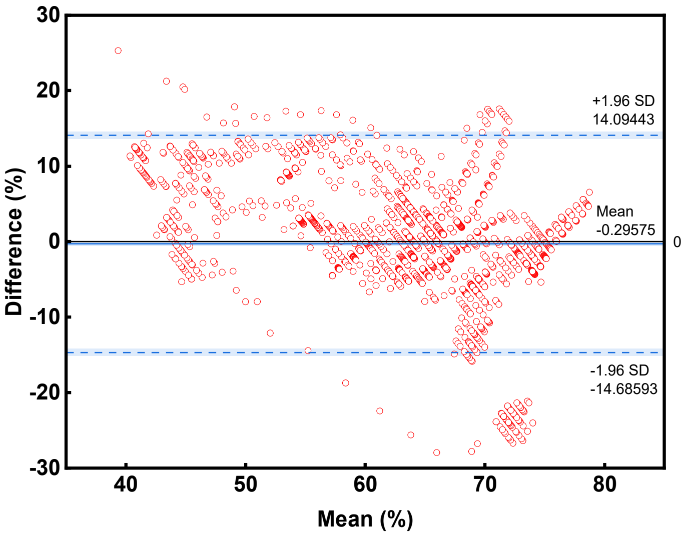

Figure S15. Bland-Altman analysis of in vivo StO_2 measurement between our device and ViOptix in porcine flap model (n=3 animals, complete event phases, 135 minutes of continuous measurement each). Source data are provided as a Source Data file.

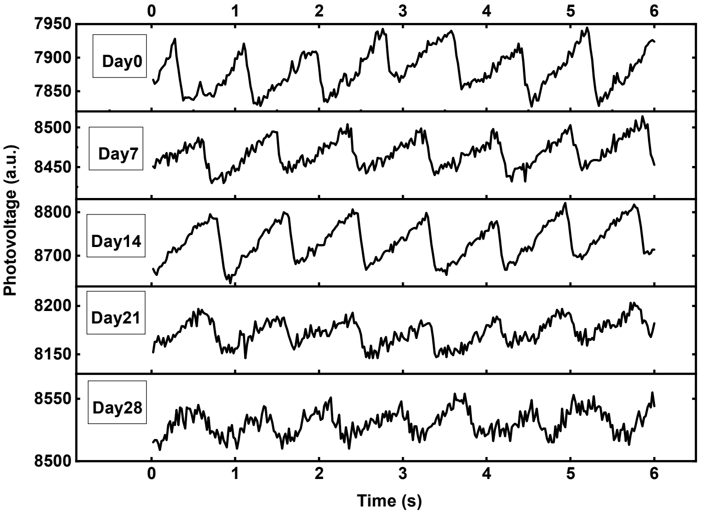

Figure S16. Measurement of pulse signals from the index finger using the NIR μ-ILED, after immersing the probe in PBS solution at 37 ℃ at various time. Source data are provided as a Source Data file.

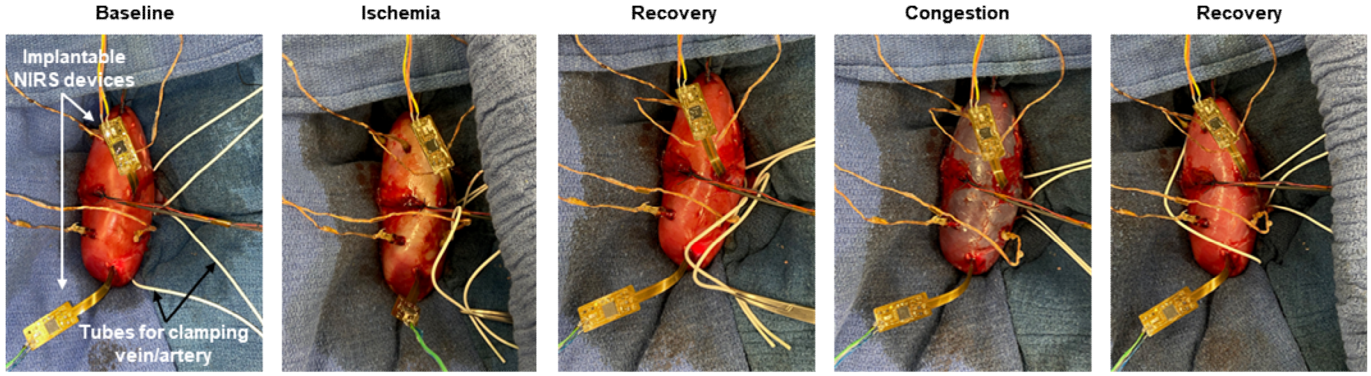

Figure S17. Images of the NIRS probe in a kidney at various conditions: baseline, ischemia, recovery, congestion, and recovery from left to right.

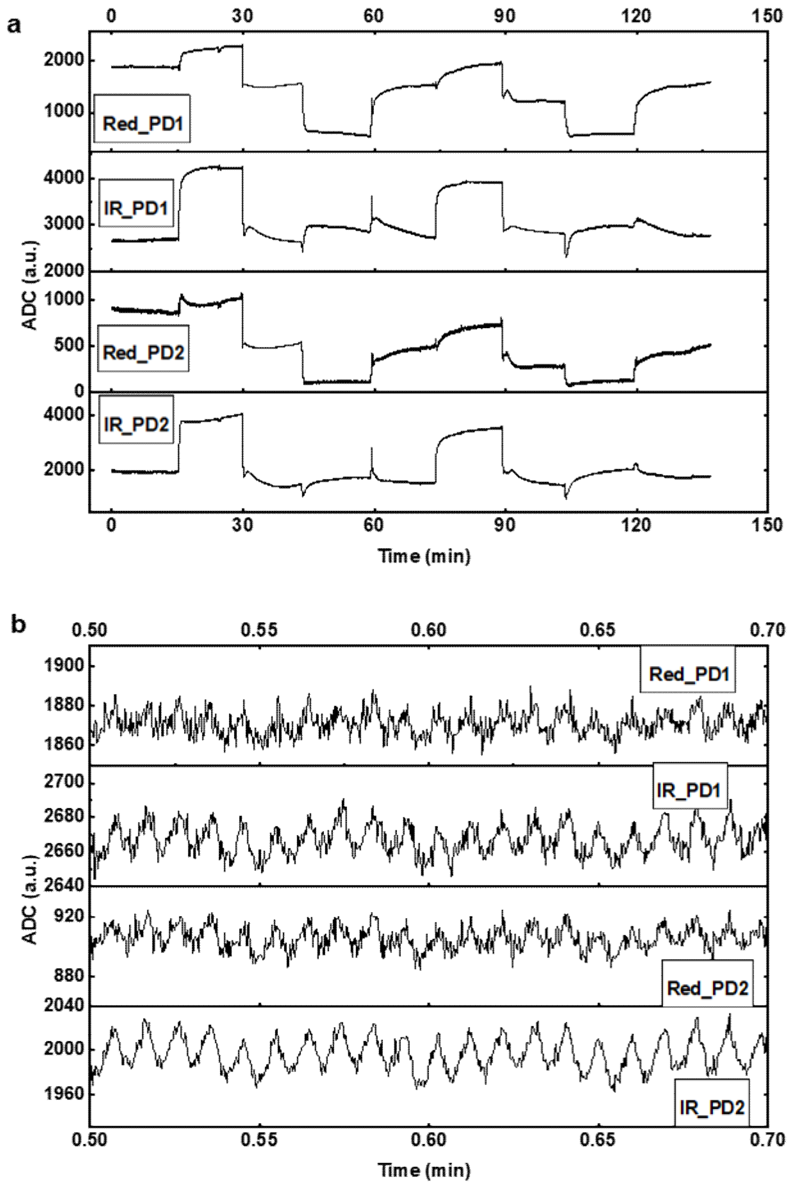

Figure S18. Measurements in a porcine kidney model. a) Representative raw data recorded from the NIRS probe in a kidney. b) Detailed view of raw data in a) showing the pulse signals. Source data are provided as a Source Data file.

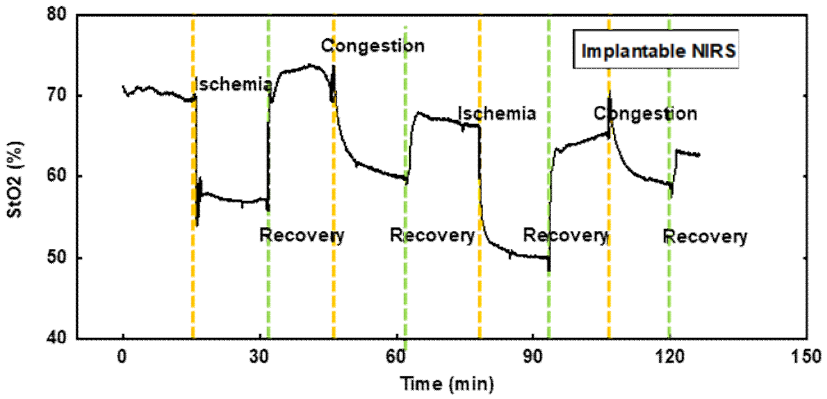

Figure S19. Additional StO_2 measurements from the NIRS probe in a porcine kidney model. Source data are provided as a Source Data file.

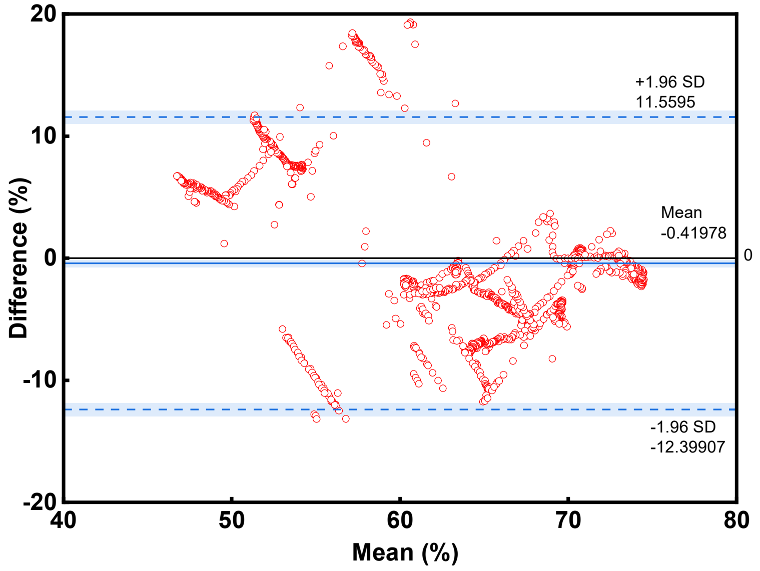

Figure S20. Bland-Altman analysis of in vivo StO_2 measurement between two devices in porcine kidney model (n=1, complete event phases, 130 minutes of continuous measurement). Source data are provided as a Source Data file.

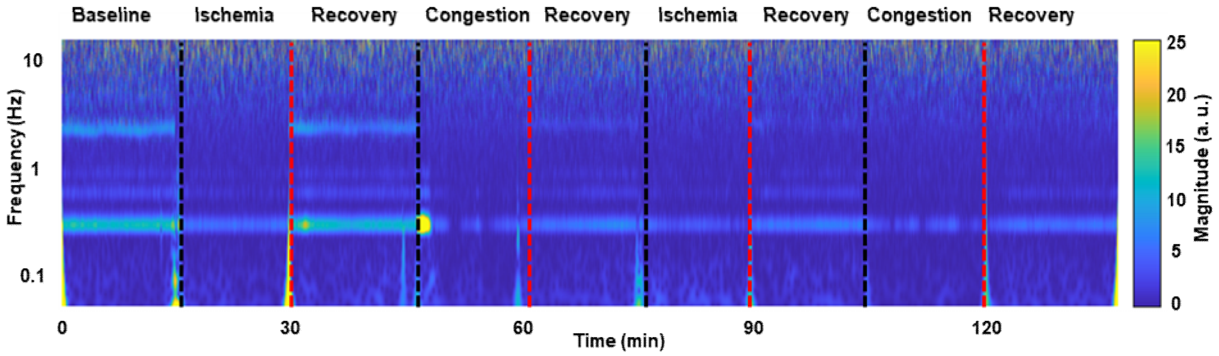

Figure S21. Frequency spectrum showing the respiratory and heart rate activity in a porcine flap model during in vivo measurements with a NIRS probe.

References

1 Bai, W. *et al.* Bioresorbable photonic devices for the spectroscopic characterization of physiological status and neural activity. *Nature biomedical engineering* **3**, 644-654 (2019).

2 Guo, H. *et al.* Advanced Materials in Wireless, Implantable Electrical Stimulators that Offer Rapid Rates of Bioresorption for Peripheral Axon Regeneration. *Advanced Functional Materials*, 2102724 (2021).

3 Shin, J. *et al.* Bioresorbable pressure sensors protected with thermally grown silicon dioxide for the monitoring of chronic diseases and healing processes. *Nature biomedical engineering* **3**, 37-46 (2019).
