# Supplementary material for: Wireless implantable optical probe for continuous monitoring of oxygen saturation in flaps and organ grafts
Source: Nat Commun. 2022 May 30;13:3009. doi: 10.1038/s41467-022-30594-z (PMC9151749; doi:10.1038/s41467-022-30594-z)
Supplement: Supplementary file 2 — Reporting Summary [file 41467_2022_30594_MOESM2_ESM.pdf]

nature portfolio

Corresponding author(s): John A. Rogers

Last updated by author(s): Mar 27, 2022

Reporting Summary

Nature Portfolio wishes to improve the reproducibility of the work that we publish. This form provides structure for consistency and transparency in reporting. For further information on Nature Portfolio policies, see our Editorial Policies and the Editorial Policy Checklist.

Statistics

For all statistical analyses, confirm that the following items are present in the figure legend, table legend, main text, or Methods section.

n/a	Confirmed	
☐	☒	The exact sample size (*n*) for each experimental group/condition, given as a discrete number and unit of measurement
☐	☒	A statement on whether measurements were taken from distinct samples or whether the same sample was measured repeatedly
☒	☐	The statistical test(s) used AND whether they are one- or two-sided<br>*Only common tests should be described solely by name; describe more complex techniques in the Methods section.*
☒	☐	A description of all covariates tested
☒	☐	A description of any assumptions or corrections, such as tests of normality and adjustment for multiple comparisons
☐	☒	A full description of the statistical parameters including central tendency (e.g. means) or other basic estimates (e.g. regression coefficient) AND variation (e.g. standard deviation) or associated estimates of uncertainty (e.g. confidence intervals)
☒	☐	For null hypothesis testing, the test statistic (e.g. *F*, *t*, *r*) with confidence intervals, effect sizes, degrees of freedom and *P* value noted<br>*Give P values as exact values whenever suitable.*
☒	☐	For Bayesian analysis, information on the choice of priors and Markov chain Monte Carlo settings
☒	☐	For hierarchical and complex designs, identification of the appropriate level for tests and full reporting of outcomes
☒	☐	Estimates of effect sizes (e.g. Cohen's *d*, Pearson's *r*), indicating how they were calculated

Our web collection on statistics for biologists contains articles on many of the points above.

Software and code

Policy information about availability of computer code

Data collection	Fig. 2e used InstrumentStudio software ((19.1.0)), available for download online from National Instruments.<br>Fig. 2f was analyzed by COMSOL 5.5.<br>Fig. 3a,b used MESUR gauge software from Mark-10 company.<br>Fig 4 and 5 used code deposited in Github. https://github.com/wouyanglv/BLE_NIRS_GUI
Data analysis	OriginPro 2021 and MATLAB R2020b were used for data analysis and plotting.

For manuscripts utilizing custom algorithms or software that are central to the research but not yet described in published literature, software must be made available to editors and reviewers. We strongly encourage code deposition in a community repository (e.g. GitHub). See the Nature Portfolio guidelines for submitting code & software for further information.

Data

Policy information about availability of data

All manuscripts must include a data availability statement. This statement should provide the following information, where applicable:

- Accession codes, unique identifiers, or web links for publicly available datasets
- A description of any restrictions on data availability
- For clinical datasets or third party data, please ensure that the statement adheres to our policy

Source data are provided with this paper.

Field-specific reporting

Please select the one below that is the best fit for your research. If you are not sure, read the appropriate sections before making your selection.

☒ Life sciences ☐ Behavioural & social sciences ☐ Ecological, evolutionary & environmental sciences

For a reference copy of the document with all sections, see nature.com/documents/nr-reporting-summary-flat.pdf

Life sciences study design

All studies must disclose on these points even when the disclosure is negative.

Sample size	The work was exploratory therefore, no sample size calculation was performed. Sample size of 3 was used for device characterization, in vivo biocompatibility of the devices, and in vivo monitoring of oxygenation. The results were consistent.
Data exclusions	No data were excluded from the analyses.
Replication	The experiments were replicated for 3 times and the results were consistent.
Randomization	The randomization is not relevant to the study because there was only one experimental group with controlled perfusion and the device was adopted to monitor the signal during this study.
Blinding	The blinding was not relevant to the study because in the porcine model, there was only one experimental group. For rat model, no blinding was necessary as there were no significant variations in the animals tested.

Reporting for specific materials, systems and methods

We require information from authors about some types of materials, experimental systems and methods used in many studies. Here, indicate whether each material, system or method listed is relevant to your study. If you are not sure if a list item applies to your research, read the appropriate section before selecting a response.

Materials & experimental systems

n/a	Involved in the study	
☒	☐	Antibodies
☒	☐	Eukaryotic cell lines
☒	☐	Palaeontology and archaeology
☐	☒	Animals and other organisms
☒	☐	Human research participants
☒	☐	Clinical data
☒	☐	Dual use research of concern

Methods

n/a	Involved in the study	
☒	☐	ChIP-seq
☒	☐	Flow cytometry
☒	☐	MRI-based neuroimaging

Animals and other organisms

Policy information about studies involving animals; ARRIVE guidelines recommended for reporting animal research

Laboratory animals	Female Sprague Dawley rat (6-8 weeks old); Male Landrace Cross pig (3-4 months old).
Wild animals	The study did not involve wild animals.
Field-collected samples	The study did not involve samples collected from field.
Ethics oversight	The Institutional Animal Care and Use Committee at Washington University, School of Medicine approved the protocol for porcine models; The Institutional Animal Care and Use Committee at Northwestern University approved protocol for rat study.

Note that full information on the approval of the study protocol must also be provided in the manuscript.
